# Enantiospecific photoresponse of sterically hindered diarylethenes for chiroptical switches and photomemories

**DOI:** 10.1038/srep09186

**Published:** 2015-03-17

**Authors:** Wenlong Li, Xin Li, Yongshu Xie, Yue Wu, Mengqi Li, Xin-Yan Wu, Wei-Hong Zhu, He Tian

**Affiliations:** 1 Key Laboratory for Advanced Materials and Institute of Fine Chemicals, Shanghai Key Laboratory of Functional Materials Chemistry, Collaborative Innovation Center for Coal Based Energy (i-CCE), East China University of Science and Technology, Shanghai 200237, China; 2 Division of Theoretical Chemistry and Biology, School of Biotechnology, KTH Royal Institute of Technology, SE-10691 Stockholm, Sweden

## Abstract

Light-driven transcription, replication and enzyme catalysis are critically dependent upon a delicate transfer between molecular and supramolecular chirality. Chemists have well realized the impressive stereospecificity over many thermally accessible cycloaddition with chiral catalysts, but making light work in the enantiomer control of diarylethene photocyclization has proved to be more challenging. Here, we report a unique sterically hindered diarylethene (BBTE) system with absolute enantiospecific photocyclization and cycloreversion. Moreover, we have fully separated all the five thermally stable isomers, consisting of one achiral parallel conformer, one pair of anti-parallel ring-open enantiomers, and another pair of ring-closed enantiomers, whose absolute chiral configurations are entirely elucidated by single X-ray crystallographic analyses. The photo-responsive feature exhibits a reversible, complete enantio-control transformation without racemism, offering an unrivaled unimolecular enantiospecific platform for potential applications as bistable chiroptical switches and all-photonic photomemories with optical rotation as non-destructive readout.

Chiral molecules and helical structures exist universally in nature, and are of vital importance to living systems^1^,^2^. Owing to the clean and precise spatiotemporal control in a remote and noninvasive manner, the light-driven chirality transformation, especially based on photochromic systems^3^,^4^, enables a myriad of potential applications in smart materials^5^,^6^,^7^,^8^,^9^ and functional bio-switches^10^,^11^,^12^ at molecular level. Amongst the most ideal candidates, diarylethenes (DAEs)^13^,^14^,^15^,^16^,^17^,^18^,^19^ undergo reversible conrotatory 6*π*-electron photocyclization and cycloreversion, along with excellent thermal irreversibility and outstanding fatigue resistance. A closer examination at the photo-switching process of DAEs reveals not only a structural change from the ring-open to the ring-closed states, but also a chirality transformation from the axial helixity of central hexatriene moiety to the central asymmetry of two reactive stereogenic centers. Since the flexible ring-open isomers show typically rapid rotation of aryl groups, the accompanied loss in inherent chirality takes place inevitably. Therefore, no racemization in the photochromic reaction between two enantiomers is highly desirable for light-driven chiroptical switches.

To deal with the dilemma, a few molecular-engineering efforts have been explored by generating intermolecular or intramolecular asymmetric environment, for instance, introducing auxiliary chiral units^20^,^21^,^22^, conducting photoreactions in the chiral crystals^23^,^24^,^25^ and supramolecular states^26^,^27^,^28^, and fixing aryl rotation through covalent bonding^29^,^30^ or steric hinderance^31^. However, these systems are always restricted with reaction conditions, such as low temperature, specific solvent dependence and limited conversion yield. Indeed, the full enantiospecific thermal bistability is still rare in DAE, and the completely isolating enantiomerically pure single crystals for absolute chiral configuration are highly desirable. Herein, we report the unique full isolation of a sterically hindered and thermally irreversible DAE (**BBTE**, Fig. 1a) to fill this blank, which consists of one achiral parallel conformer, one pair of anti-parallel ring-open enantiomers, and another pair of ring-closed enantiomers. Their corresponding absolute chiral configurations are fully confirmed by X-ray crystallographic analyses. Notably, these separated enantiomers show absolute enantiospecific photocyclization and cycloreversion both in solution and polymeric matrix. The two pairs of enantiomers are optically pure with enantiomer excess (*ee*) value over 99%, and thermally stable enough without showing any racemization. As a powerful separation of enantiomers, the mutual ability to control enantiospecific transformation at no loss in racemism can light up bistable chiroptical switches and photomemories with molecular optical rotation as non-destructive readout.

## Results and Discussion

### Full separation of five isomers based on sterically hindered benzobis(thiadiazole) ethene bridge

**Figure 1:**
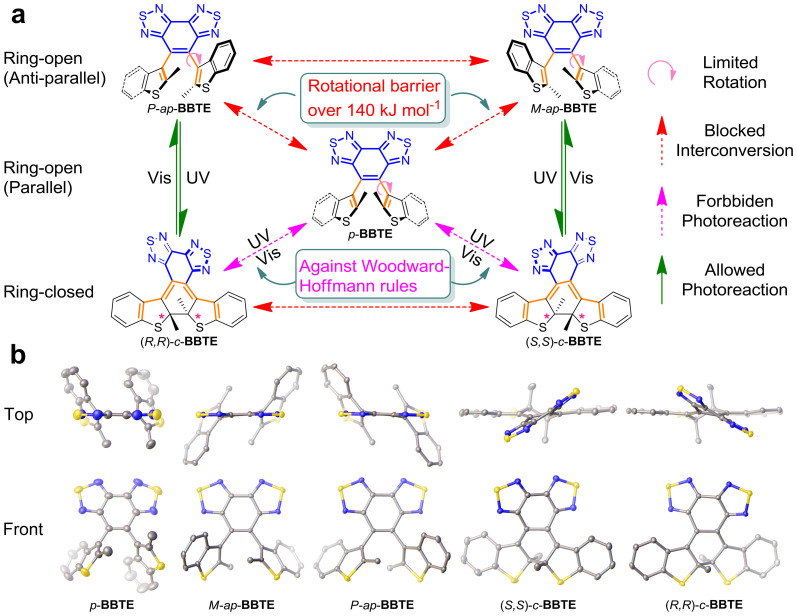
Chemical structures and isomerism of BBTE. (a) Schematic illustration of conversion relationship of five thermally stable isomers in **BBTE**. Three ring-open conformers are thermally isolated (red dashed arrows) due to extremely high rotational barrier (pink arrows). While the parallel ring-open isomer is photochemically inert (magenta dash arrows), the *anti*-parallel ring-open enantiomers are able to reversibly transform to the corresponding ring-closed enantiomers (olive arrows), according to Woodward-Hoffmann rule on photochemical conrotatory reactions of 4n + 2 π-electron system. (b) ORTEP representation of X-ray single crystal structures for *p*-, *M*-*ap*-, *P*-*ap*-, (*S,S*)-*c*-, and (*R,R*)-*c*-**BBTE** drawn with 50% probability from top and front views (S: yellow; N: blue; C: gray). The solvent molecule (dioxane) in *p*-**BBTE** and all the hydrogen atoms are removed for clarity. Atoms with lighter colours indicate their relatively back location. Detailed crystal data including packing diagrams can be found in Supplementary Information.

Ring-open (*o*-) DAEs possess a hexatriene structure whose *π*-conjugation is interrupted by the steric repulsion between two adjacent aryl rings. Common *o*-DAEs have two main conformers, photoactive *anti*-parallel (*ap*-) conformer and photo-inert parallel (*p*-) conformer (Fig. 1a), whose exchanging rate is mainly governed by the steric hindrance imposed by the two aryl rings^32^,^33^. However, in our previous work on a unique thermally bistable DAE^34^, the large bulky benzobis(thiadiazole) ethene bridge brings forth very high rotation strain, an energy barrier over 140 kJ mol^−1^ (Fig. 1a), which completely freezes the interconversion and thus results in the isolation of *anti*-parallel and parallel conformers (*ap*- and *p*-**BBTE**).

Stereochemically, compared to *p*-**BBTE** with symmetry of plane as an achiral isomer, the ring-open conformer *ap*-**BBTE** endowing *C*_2_ symmetry can be further subdivided into a pair of enantiomers with *M* and *P* helixity (Fig. 1a). It should be noted that in general ring-open diarylethenes, these two *ap*-enantiomers show rapid racemization through fast rotations of aryl groups, via *p*-conformer as an intermediate. However, because of the blocking of the transformation between *ap*- and *p*-conformers in **BBTE**, the enantiomers of *ap*-**BBTE** are also expected to be thermally isolatable. Indeed, chiral HPLC (CHIRALCEL® OD-R) chromatogram of *ap*-**BBTE** shows two clear peaks with equal area, which should belong to the two ring-open enantiomeric forms, while that of *p*-**BBTE** exhibits only single peak (Supplementary Fig. S2). Moreover, when *ap*-**BBTE** is irradiated with ultraviolet (UV) light, another pair of equal-area peaks emerge, which could be assigned as the corresponding ring-closed enantiomers of *c*-**BBTE** with two stereogenic centers.

With these in mind, we decided to separate these enantiomers on a preparative column to study their individual photochromism. The direct resolution of two ring-open *anti*-parallel enantiomers was unsuccessful on commonly used preparative chiral columns. Whereas, the two ring-closed enantiomers could be smoothly separated on Chiralpak IC column (CHIRALCEL®), and later transformed back to their corresponding ring-open enantiomers of *ap*-**BBTE** by photocycloreversion (Fig. 1a). As checked by chiral HPLC, the two pairs of enantiomers are optically pure (*ee*> 99%), and thermally stable enough without showing any racemization, even when kept in acetonitrile at 333 K for 72 h (Supplementary Fig. S3).

In contrast with the previous racemic crystals of *ap*- and *c*-**BBTE**^34^, we successfully obtained the single crystals for all the five thermally irreversible isomers (Fig. 1b), consisting of one achiral ring-open parallel conformer, one pair of anti-parallel ring-open enantiomers, and another pair of ring-closed enantiomers, by slow evaporation of mixed solvents (THF/dixoane/C_2_H_5_OH for *p*-**BBTE**, CH_2_Cl_2_/CH_3_CN for *ap*-**BBTE**, CH_2_Cl_2_/CH_3_OH for *c*-**BBTE**). Accordingly, we are able to fully determine the absolute configurations of these enantiomers by X-ray single crystal analyses. As a matter of fact, we have named them after their absolute configurations: *P*-*ap*- and *M*-*ap*-**BBTE** with axial chirality adopt *P* and *M* helicity for one pair of anti-parallel ring-open enantiomers, and (*R,R*)-*c*- and (*S,S*)-*c*-**BBTE** with two chiral carbons endow configurations as (*R*,*R*) and (*S*,*S*) for another pair of ring-closed enantiomers, respectively. It is also found that *P*-*ap*- and *M*-*ap*-**BBTE** belong to monoclinic chiral space group *C*2, and (*R,R*)-*c*- and (*S,S*)-*c*-**BBTE** to orthorhombic chiral space group *P*2_1_2_1_2_1_ (Supplementary Fig. S9–S12 and Table S2, S3). *p*-**BBTE** lies in triclinic achiral space group *P*-1^34^.

### Reversibly enantiospecific transformation between photocyclization and cycloreversion with no racemization

**Figure 2:**
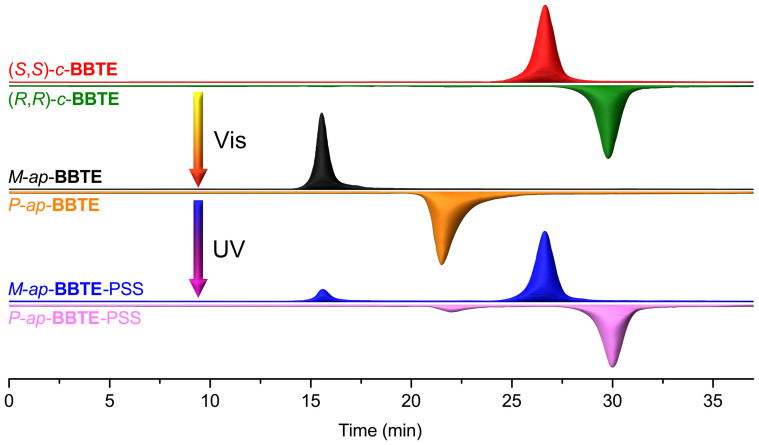
HPLC chromatogram of light-driven enantiospecific transformation of *c*-BBTE and *ap*-BBTE in CH_3_CN. (*R,R*)- and (*S,S*)-*c*-**BBTE** were bleached with visible light (*λ* > 470 nm) to generate *P*- and *M*-*ap*-**BBTE**, and then irradiated with UV light (*λ* = 280 nm) to reach PSS, respectively. It is noted that (*R,R*)-*c*-**BBTE**, *P*-*ap*-**BBTE**, and PSS of *P*-*ap*-**BBTE** are vertically flipped for better comparison. Reversibly enantiospecific photochromism between photocyclization and cycloreversion can only proceed in [(*S,S*)-*c*-**BBTE** and *M*-*ap*-**BBTE**], and [(*R,R*)-*c*-**BBTE** and *P*-*ap*-**BBTE**]. Column: OD-R (CHIRALCEL® 4.6 diameter × 250 mm); Flow rate: 0.8 mL min^−1^; eluent: CH_3_CN/H_2_O (80/20, *v*/*v*); detecting wavelength: 303 nm (isobestic point).

We first employed chiral HPLC (CHIRALCEL® OD-R, reversed phase) to monitor the photochromic reactions (Fig. 2). Interestingly, both (*S,S*)-*c*-**BBTE** and *M*-*ap*-**BBTE** show shorter retention time than (*R,R*)-*c*-**BBTE** and *P*-*ap*-**BBTE**, correspondingly, which may be ascribed to the relative weaker affinity of *M*-helixity or similar structure (*S*,*S* configuration) with the cellulose coating on the chiral column. When irradiating the acetonitrile solution of (*S,S*)-*c*-**BBTE** with broadband visible (Vis) light (*λ* > 470 nm), a peak belonging to *M*-*ap*-**BBTE** is exclusively generated in the chromatogram. Upon irradiation of the above solution with UV light at 280 nm until the photostationary state (PSS), the peak of *M*-*ap*-**BBTE** is decreased and exclusively converted to that of (*S,S*)-*c*-**BBTE** again. Arguably, from the HPLC chromatograms, it is unambiguous that the photochromic reactions proceed enantiospecifically and reversibly in two groups as [(*S,S*)-*c*-**BBTE** and *M*-*ap*-**BBTE**], and [(*R,R*)-*c*-**BBTE** and *P*-*ap*-**BBTE**].

**Figure 3:**
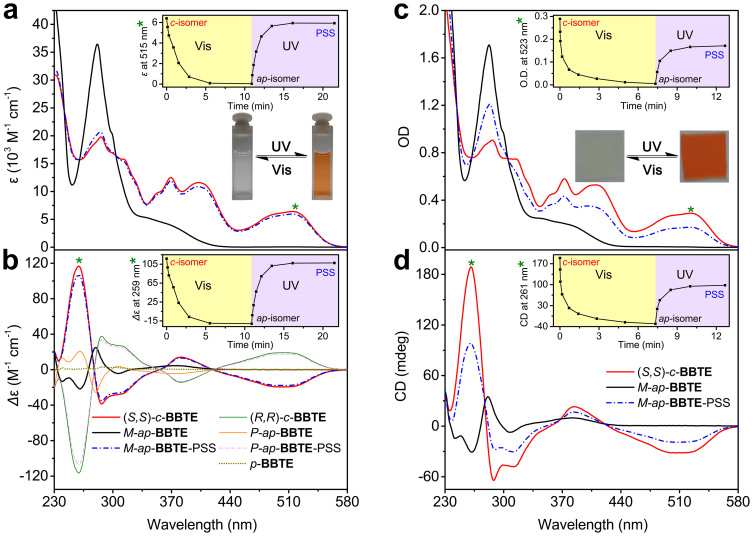
Light-induced spectral changes in enantiospecific photoreactions of *c*-BBTE and *ap*-BBTE. Absorption (a) and CD spectra (b) of (*S,S*)-*c*-**BBTE**, *M*-*ap*-**BBTE**, and PSS of *M*-*ap*-**BBTE** under irradiation of UV light (*λ* = 280 nm) in CH_3_CN. Absorption (c) and CD spectra (d) of (*S,S*)-*c*-**BBTE**, *M*-*ap*-**BBTE**, and PSS of *M*-*ap*-**BBTE** under irradiation of UV light (*λ* = 302 ± 20 nm) in poly(*D/L*-lactic acid) film (PDLLA, *M*_w_ = 2.5 × 10^5^, 1.0 wt%, 35 μm in thickness). (*R,R*)-*c*-**BBTE**, *P*-*ap*-**BBTE**, and *p*-**BBTE** are also presented in b for comparison. Insets in a and b: spectral changes of absorption at 515 nm (a) and CD at 259 nm (b) upon irradiation of (*S,S*)-*c*-**BBTE** with visible light (*λ* > 470 nm) and then UV light (*λ* = 280 nm). Insets in c and d: spectral changes of absorption at 523 nm and CD changes at 261 nm upon irradiation of (*S,S*)-*c*-**BBTE** with visible light (*λ* > 470 nm) and then UV light (*λ* = 302 ± 20 nm). Photos in a and c show the colour changes upon irradiation with UV and visible light, alternatively. OD represents ‘optical density'. The cycloreversion under visible light is fully complete, and the photocyclization under UV light is incomplete, with conversion ratios of 92% in CH_3_CN and 59% in PDLLA, calculated from absorption spectra.

As shown in Fig. 3a, upon irradiation of visible light (*λ* > 470 nm), the orange red acetonitrile solution of (*S,S*)-*c*-**BBTE** turned colourless, along with a decrease at 515, 403, and 369 nm, and an increase at 282 nm in the absorption spectra, due to the disassembly of π-conjugation of cyclohexadiene structure. Ultimately, the visible absorption is lost completely to yield a colourless solution, indicative of the full cycloreversion to *M*-*ap*-**BBTE**. Upon UV irradiation at 280 nm, the resultant colourless solution became orange red again, closely similar to the original absorption of (*S,S*)-*c*-**BBTE** (Fig. 3a and inset), giving rise to a photocyclization conversion ratio of 92% at PSS (calculated from absorption spectra). In consistent with previous report on racemic *ap*-**BBTE**^34^, chiral *M*-*ap*-**BBTE** indeed exhibits a relative higher photocyclization quantum yield (*Φ*_o–c_ = 73%) than that of common DAEs (*Φ*_o–c_ ≤ 50%), thus guaranteeing the higher cyclization conversion ratio and the fast colouring speed in solution (Table 1). Similar phenomenon and properties are also observed in another group of enantiomers (*R,R*)-*c*-**BBTE** and *P*-*ap*-**BBTE** (Supplementary Fig. S4).

### Light-driven control in circular dichroism (CD)

Since the two pairs of photo-responsive enantiomers are fully separated and reversibly enantiospecific, we shed much light on CD modulation between photocyclization and cycloreversion. As expected, the CD spectra of (*R,R*)-*c*- and (*S,S*)-*c*-**BBTE**, as well as *P*-*ap*- and *M*-*ap*-**BBTE**, show exact mirror images, and thus essentially testifying their enantiotopic nature (Fig. 3b). CD spectrum of (*S,S*)-*c*-**BBTE** exhibits moderately negative Cotton effect in the visible region of 420–580 nm, with the same orientation and relative larger intensity than the reported *c*-DAEs with (*S,S*) configuration^35^,^36^. It can be assigned to the charge transfer excitation of HOMO → LUMO. Other signals in 353–420 nm (+) and 278–353 nm (−) can also be attributed to the charge transfer excitation. Interestingly, the ring-closed (*S,S*)-*c*-**BBTE** endows an abnormally strong peak at 259 nm (*Δε* = 116.8 M^−1^ cm^−1^), much higher than common *c*-DAE (less than 25 M^−1^ cm^−1^)^21^,^22^,^29^, even close to those famous overcrowded alkenes^37^ and binaphthyls^38^,^39^ with axial chirality. TDDFT calculations (Supplementary Fig. S6–8) demonstrate that there is a high asymmetry in (*S,S*)-*c*-**BBTE**, originating from the photochromic cyclohexadiene center (Fig. 1a, orange unit) and the distorted ethene bridge with two extended thiadiazoles (Fig. 1a, blue unit), and the local excitations of the later mainly contributes to this strong signal in far-UV region. Nonetheless, the axially chiral ring-open *M*-*ap*-**BBTE** also displays several weaker Cotton effects in the near-UV region of 321–450 nm (+) and 297–321 nm (−), belonging to the charge transfer excitation, and those in the far-UV regions of 271–297 nm (+) and 234–271 nm (−) are originated from local excitations of the benzothiophene units. Upon irradiation of the solution of ring-closed (*S,S*)-*c*-**BBTE** with visible light (*λ* > 470 nm), the CD spectra gradually changed to that of *M*-*ap*-**BBTE**. When later irradiating the bleached solution with UV light at 280 nm, the CD spectra once again turned similar to that of (*S,S*)-*c*-**BBTE** (Fig. 3b and inset), with a conversion ratio of 92% (calculated from CD spectra). Obviously, the chirality of ring-closed isomer *c*-**BBTE** containing the asymmetric cyclohexadiene and the distorted ethene bridge can be effectively transferred to the helixity of ring-open isomer *ap*-**BBTE** from the central hexatriene moiety (*vide infra*). Impressively, several photo-switching cycles observed by CD spectroscopy show that little racemization takes place during the photocyclization and cycloreversion (Supplementary Fig. S5). Again, the unique **BBTE** based on hindered benzobis(thiadiazole)-based ethene bridge induces very high rotation strain to completely freeze the large bulky terminal benzothiophene with no racemization. Here the established reversible light-driven CD modulation provides an all-photonic enantiospecific molecular building block.

### Enabling chirality for bistable chiroptical switches and nondestructive readout

Generally for photochromic DAEs, achieving the non-destructive storage^40^,^41^,^42^,^43^,^44^ is always a long standing challenge since reading photomemory by absorption spectral changes inevitably induces the molecular photo-excitation, thus suffering from the data loss after certain times of accumulation. However, employing output signals from optical rotation can fundamentally solve the problem^30^,^45^,^46^. In this way, we can detect the light polarization in the region outside their electronic absorption bands (no absorption at this wavelength for ring-open and closed isomers), thus eliminating any photo-excitation induced structural changes. As photomemory materials, the enantiospecific transformation between photocyclization and cycloreversion is well reversibly light-modulated with no racemization, being a delicate molecular platform for chiroptical switches and nondestructive readout. Thus, we attempted to measure the optical rotation of the two isolated pairs of enantiomers of *ap*-**BBTE** and *c*-**BBTE** in acetonitrile solution at 633 nm, where both *ap*-**BBTE** (*λ* < 440 nm) and *c*-**BBTE** (*λ* < 580 nm) are transparent with no absorption. The specific rotation of (*R,R*)-*c*- and (*S,S*)-*c*-**BBTE** has almost the same absolute value with opposite signs, as well as that of *P*-*ap*- and *M*-*ap*-**BBTE** (Table 1). Exactly, the photochromic reaction can also be monitored by specific rotation changes. For instance, *M*-*ap*-**BBTE** exhibited a relatively small value of +424° at 280 nm, while the value decreased to −1891° at PSS, which was close to that of (*S,S*)-*c*-**BBTE** (−2109°). The optical rotation brought forth a large change of −2315°, indicative of a photocyclization conversion of 91%, consistent with the calculation from absorption and CD spectra. Furthermore, these optical rotation values show little changes upon long time placing in the polarimeter with the detecting light (optical filter at 633 nm), suggestive of the promising non-destructive readout capability.

**Figure 4:**
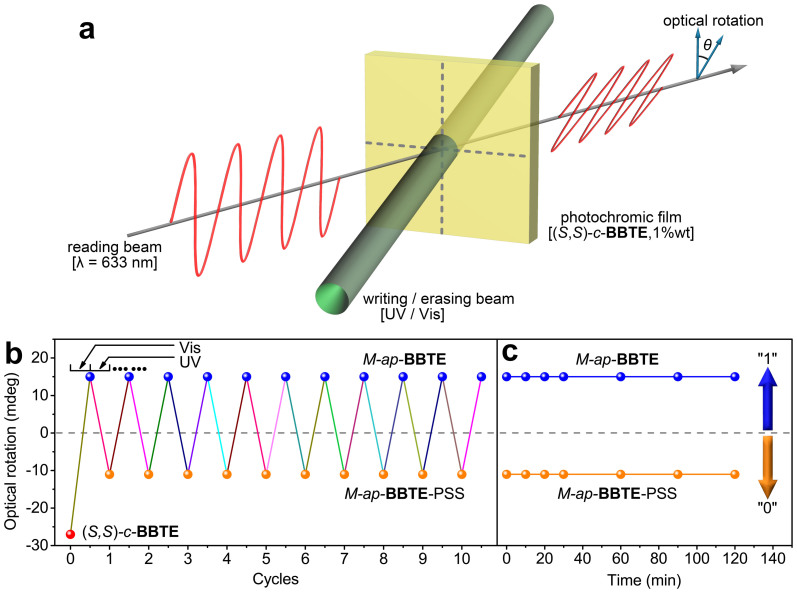
Non-destructive readout on photochromic film utilizing optical rotation as output signal for photomemories. (a) Schematic illustration of an all-photonic memory device with non-destructive readout capability. UV (*λ* = 302 ± 20 nm) and visible (*λ* > 470 nm) light is used for writing and erasing processes, while detecting optical rotation is selected for reading process, utilizing polarized light (*λ* = 633 nm). Photochromic film: PDLLA film 60 (μm) containing 1.0 wt% of (*S,S*)-*c*-**BBTE**. (b) Fatigue resistance of optical rotation upon irradiation of visible light (*λ* > 470 nm) and UV light (*λ* = 302 ± 20 nm), alternatively. (c) Photo-stability of optical rotation under the irradiation at 633 nm (1.5 mW cm^−2^), optically filtered from a white LED (3 W). Setting zero point as the threshold for “0” and “1” states can construct an all-photonic molecular binary storage device with excellent fatigue resistance and non-destructive readout capability.

Inspired by these results and in order to further realize practical applications, we fabricated several photochromic films (Fig. 4a), containing 1.0 wt% of (*S,S*)-*c*-**BBTE** in poly(*D/L*-lactic acid) (PDLLA, *M*_w_ = 2.5 × 10^5^). The film (35 μm) showed similar absorption and CD spectra (Fig. 3c–d) with respect to the solution state. In the PDLLA polymeric matrix, its absorption shows slight bathochromic shift (*λ*_max_ = 523 nm), which can be ascribed to the different polarity in PDLLA matrix, but its absorption edge is still below 600 nm. The orange-red film show excellent cycloreversion with complete bleaching upon irradiation of visible light (*λ* > 470 nm), but the recolouring progress with UV light (hand-held lamp, *λ* = 302 ± 20 nm) was not so robust with respect to that in solution, only inducing a photocyclization ratio of 60%, which is commonly found in the films of DAEs^44^,^47^,^48^, mainly due to the inner filter effects and restricted reaction environment in the polymer matrixes.

The optical rotation of another thicker film (60 μm) at 633 nm also shows full cycloreversion and partial photocyclization (62%). Fortunately, despite the first “bleaching/colouring” cycle, the following cycles could be stabilized at the specific ratio between ring-open and PSS states, ensuring the fatigue resistance in the polymeric film. As illustrated with a pair of enantiomers [(*S,S*)-*c*-**BBTE** and *M*-*ap*-**BBTE**], the optical rotation value between the two states could maintain almost same even after 10 cycles (Fig. 4b). Furthermore, these values exhibit negligible change even when the film was exposed to the strong light at 633 nm (1.5 mW/cm^2^) for 120 min (Fig. 4c). Apparently, the reading process of optical rotation with the polarized light (*λ* = 633 nm) is insensitive to both the ring-open and closed forms, thus eliminating the possible destruction during the writing (UV light, *λ* = 302 ± 20 nm) and erasing (visible light, *λ* > 470 nm). Indeed, the PDLLA polymer film with two pairs of **BBTE** enantiomers can achieve the excellent non-destructive readout ability. As shown in Fig. 4a, an all-photonic molecular binary storage device can be constructed with setting zero point of light-driven optical rotation as the threshold for “0” and “1” states (Fig. 4c), along with excellent fatigue resistance and non-destructive readout capability. More practically, measuring optical rotation value can be further simplified by detecting the intensity of the output light through a polarizer for zero calibration. In this way, the information data are coming on-stream, which can be read on an “on-off” manner based on Malus' law (*I* = *I*_0_cos^2^*θ*). In contrast with common light-driven molecular logic gates in solution^49^, the incorporation into polymer matrix can provide a route toward all solid-state systems for fabricating a layer of all-photonic logic^50^, especially in encoding optical signals^51^ and transistor sensors^52^.

## Conclusions

A sterically hindered diarylethene (**BBTE**) has been designed for absolute enantiospecific photocyclization and cycloreversion. In this unique bistable system, we have unprecedentedly separated all the five thermally irreversible isomers of diarylethenes, consisting of one achiral parallel conformer (*p*-**BBTE**), one pair of anti-parallel ring-open enantiomers (*P*-*ap*- and *M*-*ap*-**BBTE**), and another pair of ring-closed enantiomers [(*R,R*)-*c*- and (*S,S*)-*c*-**BBTE**]. All the absolute configurations of these isomers are entirely characterized by single crystal X-ray analyses. The intrinsic racemism in common diarylethenes is fundamentally solved, lightening up inner chiral response to light stimulus. As exemplified by the light-driven optical rotation studies in polymeric matrix, we provide a route toward all solid-state systems for fabricating a layer of all-photonic logic. The reversibly and precisely light-driven control in circular dichroism and optical rotation provides a powerful molecular platform for bistable chiroptical switches and photomemories with non-destructive readout capability.

## Methods

### General

NMR spectra were recorded using Bruker AM-400 spectrometers. CDCl_3_ were used as solvents with tetramethylsilane (TMS) as an internal reference. High resolution mass spectra (HRMS) were carried our with a Waters LCT Premier XE spectrometer. HPLC analyses were performed by using an Agilent 1100 instrument equipped with CHIRALCEL® OD-R column (4.6 diameter × 250 mm), at flow rate of 0.8 mL min^−1^, eluent solvents of CH_3_CN/H_2_O (80/20, v/v), detecting at isobestic point wavelength of 303 nm. Absorption and CD spectra were recorded using Agilent Cary 60 and Jasco J-819 spectropolarimeter, respectively. Optical rotation values were determined with a Rudolph Autopol V polarimeter, equipped with an interference filter at 633 nm, containing a 100 mm flow cell at 293 K. Solvents used were analytical grade, except those for optical tests, which were HPLC grade. The photochromic reaction was induced by continuous irradiation using an Hg/Xe lamp (Hamamatsu, LC8 Lightningcure, 200 W) or a white LED (3 W) equipped with a narrow band interference filters for *λ*_irr_ = 280 nm, or a broad band interference filters for *λ*_irr_ > 470 nm, except for the photocyclization in the PDLLA film which was induced by a hand-held UV lamp (0.12 mW cm^−2^, *λ* = 302 ± 20 nm) for irradiation.

### Full enantiomer separation of BBTE

A pair of ring-closed enantiomers [(*R,R*)-*c*- and (*S,S*)-*c*-**BBTE**] was separated from the racemic *c*-**BBTE** by a preparative HPLC equipped with CHIRALCEL® Chiralpak IC (50 diameter × 250 mm, eluent: dichloromethane/*n*-hexane = 4/6, *v*/*v*). Another pair of ring-open enantiomers (*P*-*ap*- and *M*-*ap*-**BBTE**) were obtained by irradiating (*R,R*)-*c*- and (*S,S*)-*c*-**BBTE** with visible light (*λ* > 470 nm) in dichloromethane, and then recrystallization from dichloromethane/*n*-hexane, respectively. (*R,R*)-*c*-**BBTE**:^1^H NMR (400 MHz, CDCl_3_, ppm): *δ* 2.01 (s, 6 H), 6.98–7.07 (m, 2 H), 7.29 (d, *J* = 3.9 Hz, 4 H), 8.00 (d, *J* = 8.2 Hz, 2 H). HRMS (ESI positive ion mode for [M + H]^+^): Calcd for C_24_H_15_N_4_S_4_, 487.0180; found, 487.0183. [α]_633_^25^ = +2095° (*c* = 1.76 × 10^−2^ g dL^−1^ in CH_3_CN). (*S,S*)-*c*-**BBTE**: ^1^H NMR (400 MHz, CDCl_3_, ppm): *δ* 2.01 (s, 6 H), 6.99–7.07 (m, 2 H), 7.29 (d, *J* = 3.9 Hz, 4 H), 8.00 (d, *J* = 8.1 Hz, 2 H). HRMS (ESI positive ion mode for [M + H]^+^): Calcd for C_24_H_15_N_4_S_4_, 487.0180; found, 487.0179. [α]_633_^25^ = −2109° (*c* = 1.84 × 10^−2^ g dL^−1^ in CH_3_CN). *P*-*ap*-**BBTE**: ^1^H NMR (400 MHz, CDCl_3_, ppm): *δ* 1.94 (s, 6 H), 7.11 (d, *J* = 8.0 Hz, 2 H), 7.16–7.23 (m, 2 H), 7.23–7.30 (m, 2 H, overlap with CHCl_3_), 7.74 (d, *J* = 7.9 Hz, 2 H). HRMS (ESI positive ion mode for [M + H]^+^): Calcd for C_24_H_15_N_4_S_4_, 487.0180; found, 487.0175. [α]_633_^25^ = −420° (*c* = 1.76 × 10^−2^ g dL^−1^ in CH_3_CN). *M*-*ap*-**BBTE**: ^1^H NMR (400 MHz, CDCl_3_, ppm): *δ* 1.94 (s, 6 H), 7.11 (d, *J* = 8.0 Hz, 2 H), 7.16–7.23 (m, 2 H), 7.23–7.31 (m, 2 H, overlap with CHCl_3_), 7.74 (d, *J* = 7.9 Hz, 2 H). HRMS (ESI positive ion mode for [M + H]^+^): Calcd for C_24_H_15_N_4_S_4_, 487.0180; found, 487.0177. [α]_633_^25^ = +424° (*c* = 1.84 × 10^−2^ g dL^−1^ in CH_3_CN).

### Single crystal X-ray structure determination

The crystallographic data reported in this article have been deposited at the Cambridge Crystallographic Data Centre (CCDC), under deposition number 1016420 for (*S,S*)-*c*-**BBTE**, 1016419 for (*R,R*)-*c*-**BBTE**, 1016417 for *M*-*ap*-**BBTE**, 1016418 for *P*-*ap*-**BBTE**, and 972736 for *p*-**BBTE**. These data can be obtained free of charge form http://www.ccdc.cam.ac.uk/data_request/cif. Detailed crystallographic analyses are given in the Supplementary Information.

### Preparation of photochromic film

The photochromic film was prepared under dark conditions as follows: 2.1 mg (1.0 wt%) of (*S,S*)-*c*-**BBTE** and 203.5 mg (99.0 wt%) of PDLLA (*M*_w_ = 2.5 × 10^5^) were dissolved in 2.0 mL of dichloromethane. The solution was filtered by a filtering membrane (0.22 μm) before being spin-coated on quartz plates using a spin-coater. After air drying for 24 h, several films with different thickness (35–80 μm) were thus obtained.

## Author Contributions

W.Z., H.T. and W.L. conceived the experiments and designed the study. W.L. carried out the synthesis and chiral separation. W.L., Y.W., M.L. and X.W. performed the optical experiments. X.L. did the quantum chemical calculations. W.L. and Y.X. worked on X-ray single crystal analysis. W.Z. and W.L. wrote manuscript. All authors discussed results and contributed to the interpretation of data.

## Supplementary Material

Supplementary Information Enantiospecific photoresponse of sterically hindered diarylethenes for chiroptical switches and photomemories

## Figures and Tables

**Table 1:** Optical data of (*R,R*)-*c*-, (*S,S*)-*c*-, *P*-*ap*-, and *M*-*ap*-BBTE in CH_3_CN at 293 K

Compound	*λ*_OD,max_[a] (nm) [*ε* (10^3^ M^−1^ cm^−1^)]	*λ*_CD,max_[a] (nm) [*Δε* (M^−1^ cm^−1^)]	*Φ*_o-c_[b] (%)	*Φ*_c-o_[b] (%)	*CR*[c] (%)	[*α*]_633_[d] (°) [PSS at 280 nm]
*M*-*ap*-BBTE	282 [36.4]	280 [+24.8]	73	—	92	+424 [−1891]
(*S,S*)-*c*-BBTE	515 [6.39]	503 [−19.4]	—	6.0	>99	−2109
*P*-*ap*-BBTE	282 [35.6]	280 [−25.5]	72	—	92	−420 [+1881]
(*R,R*)-*c*-BBTE	515 [6.32]	503 [+19.1]	—	5.8	>99	+2095

[a] Typical absorption and CD maxima of ring-open isomer in UV region and ring-closed isomer in visible region, respectively. OD represents ‘optical density'.

[b] Quantum yields of photocyclization at 313 nm and photocycloreversion at 517 nm, with uncertainty around ±5% and ±0.5%, respectively.

[c] Conversion ratio to ring-closed isomer (under UV irradiation, *λ* = 280 nm), and ring-open isomer (under visible light irradiation, *λ* > 470 nm), calculated from absorption spectra.

[d] Specific optical rotation at 633 nm, outside the absorption region (*λ* < 580 nm) of both ring-open and ring-closed isomers.
