# Supplementary material for: Enantiospecific photoresponse of sterically hindered diarylethenes for chiroptical switches and photomemories
Source: Sci Rep. 2015 Mar 17;5:9186. doi: 10.1038/srep09186 (PMC4361853; doi:10.1038/srep09186)
Supplement: Supplementary Information — Enantiospecific photoresponse of sterically hindered diarylethenes for chiroptical switches and photomemories [file srep09186-s1.doc]

**Supplementary Information**

**Enantiospecific photoresponse of sterically hindered diarylethenes for chiroptical switches and photomemories**

Wenlong Li^1^, Xin Li^2^, Yongshu Xie^1^, Yue Wu^1^, Mengqi Li^1^, Xin-Yan Wu^1^, Wei-Hong Zhu^1^ and He Tian^1^

^1^Key Laboratory for Advanced Materials and Institute of Fine Chemicals, Shanghai Key Laboratory of Functional Materials Chemistry, Collaborative Innovation Center for Coal Based Energy (i-CCE), East China University of Science and Technology, Shanghai 200237, China, ^2^Division of Theoretical Chemistry and Biology, School of Biotechnology, KTH Royal Institute of Technology, SE-10691 Stockholm, Sweden.

Correspondence and requests for materials should be addressed to W.-H.Z. (email: whzhu@ecust.edu.cn).

**Contents**

[1 Chiral resolution 2](#__RefHeading___Toc395905471)

[2 HPLC study 3](#__RefHeading___Toc395905472)

[3 Supplementary spectra 4](#__RefHeading___Toc395905473)

[4 Quantum chemical calculation 5](#__RefHeading___Toc395905474)

[5 Crystal data 8](#__RefHeading___Toc395905475)

[6 Characterization 12](#__RefHeading___Toc395905476)

# 1. Chiral resolution


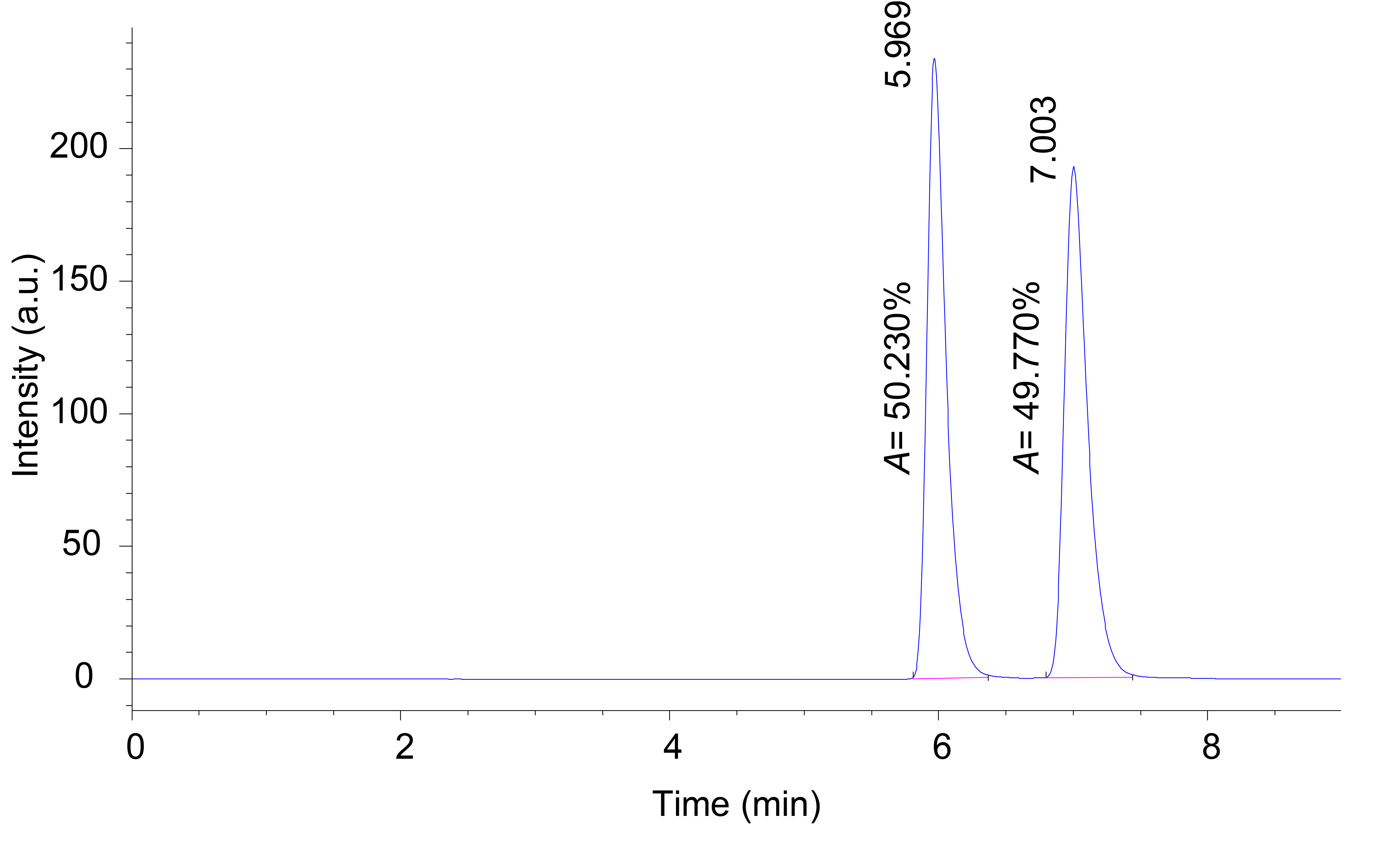


**Figure S1.** HPLC chromatograms (CHIRALCEL® Chiralpak IC, 4.6 diameter × 250 mm) of racemic *c*-**BBTE**, eluted with dichloromethane/n-hexane = 40:60, at a flow rate of 1.0 mL min^-1^ detected at 254 nm.

# 2. HPLC study

**Figure S2.** HPLC chromatograms (CHIRALCEL® OD-R, 250 diameter × 4.6 mm) of *p*-, *ap*-, and *c*-**BBTE**, eluted with CH_3_CN/H_2_O = 80:20 at a flow rate of 0.8 mL min^-1^, detected at 303 nm (isobestic point).

**Figure S3.** Thermal stabilities of (*R,R*)- and (*S,S*)-*c*-**BBTE**, *P*-*ap*- and *M*-*ap*-**BBTE** in CH_3_CN at 333 K for 72 h, monitored by HPLC (CHIRALCEL® OD-R). No thermal racemism could be found after 72 h.

# 3. Supplementary spectra

**Figure S4**. Absorption spectra of (*R,R*)-*c*-**BBTE**, *P*-*ap*-**BBTE**, and the PSS of *P*-*ap*-**BBTE** under irradiation of UV light (*λ* = 280 nm) in CH_3_CN.

**Figure 5.** Fatigue resistance of CD signals of (*S,S*)-*c*-**BBTE** in CH_3_CN upon irradiation of visible light (*λ* > 470 nm) and UV light (*λ* = 280 nm), alternatively.

# 4. Quantum chemical calculation

Based on the most stable conformations of *P*-*ap*-**BBTE** and (*R*,*R*)-*c*-**BBTE**, we computed their absorption and circular dichroism spectra by means of TDDFT calculations, as shown in **Table S1** and **Figure S6**. In **Table S1**, the molecular orbitals located on the benzobis(thiadiazole) bridge are marked in red such that the charge-transfer excitations and local excitations could be distinguished.

For *P*-*ap*-**BBTE**, the low-lying excited states S_1_, S_3_ and S_6_ correspond to charge transfer excitations from the benzothiophene units to the benzobis(thiadiazole) bridge, while S_4_ and S_9_ with large oscillator strengths correspond to local excitations of the benzobis(thiadiazole) bridge. After photo-induced cyclization of *P*-*ap*-**BBTE**, new absorption bands at near-UV and visible region arise, which are dominantly contributed by charge transfer excitations from the photochromic cyclohexadiene backbone to the benzobis(thiadiazole) bridge. Local excitations of the benzobis(thiadiazole) bridge at around 270 nm exhibit smaller oscillator strengths compared with those of the open-ring isomer. The computed absorption spectra have nicely reproduced the experimental observation and provide explanation for the emerging bands at 330−580 nm and the diminished bands at around 270 nm.

The computed CD spectra of *P*-*ap*-**BBTE** and (*R*,*R*)-*c*-**BBTE** also show excellent agreement with experimental results. For *P*-*ap*-**BBTE**, charge transfer excitations (S_1_ and S_6_) contribute to the weak Cotton effects at near-UV region, while the strong Cotton effects mainly originate from local excitations of the benzothiophene units (S_10_ and S_16_). After photocyclization, a new positive Cotton band arises at the visible region owing to the HOMO→LUMO charge transfer excitation of (*R*,*R*)-*c*-**BBTE**. The negative Cotton band at around 370 nm is enhanced, which is also attributed to charge transfer excitations. Different from *P*-*ap*-**BBTE**, the very strong CD bands of (*R*,*R*)-*c*-**BBTE** at far-UV region (S_8_ and S_19_) mainly originate from local excitations of the benzobis(thiadiazole) bridge owing to its twisted conformation.


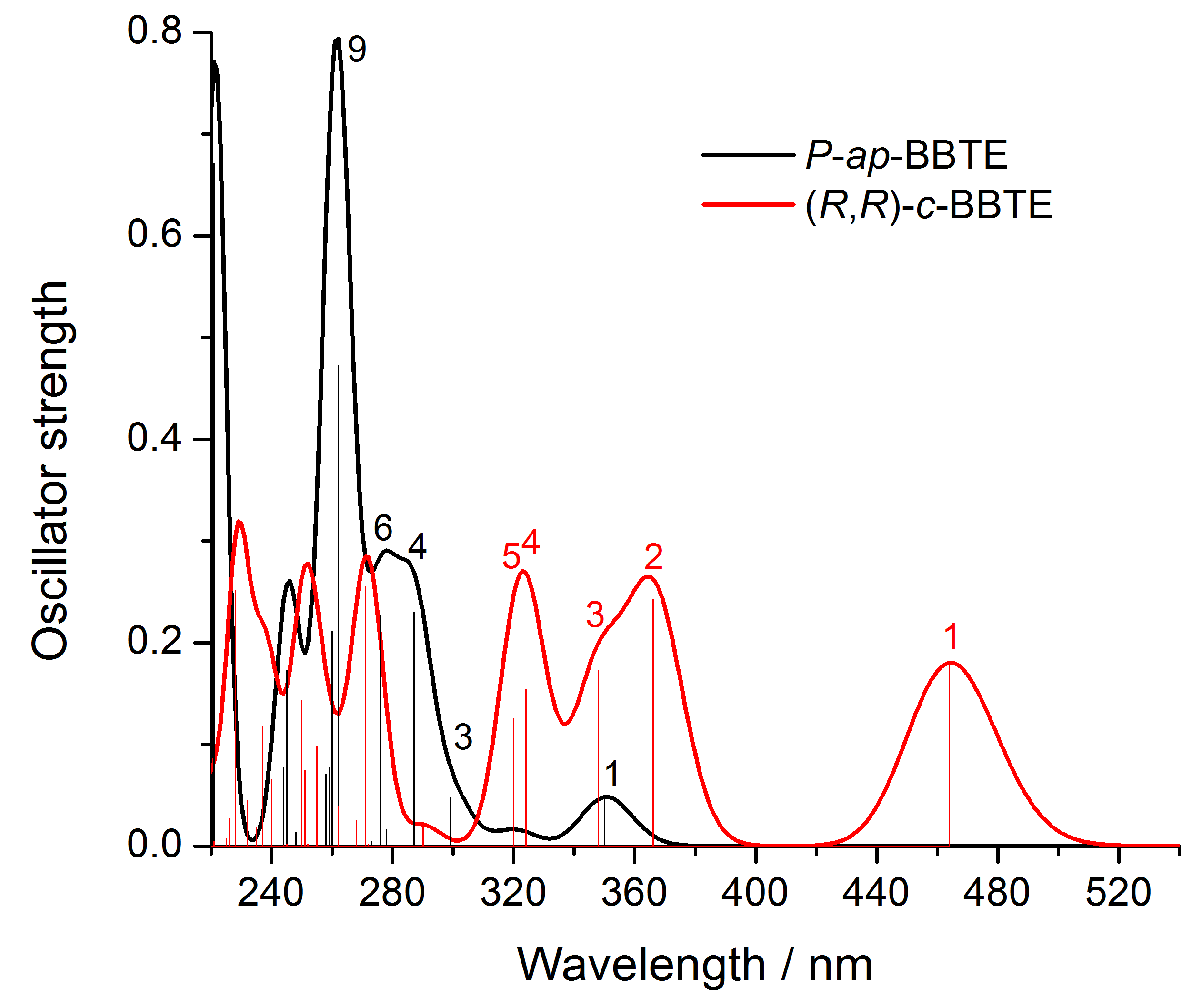

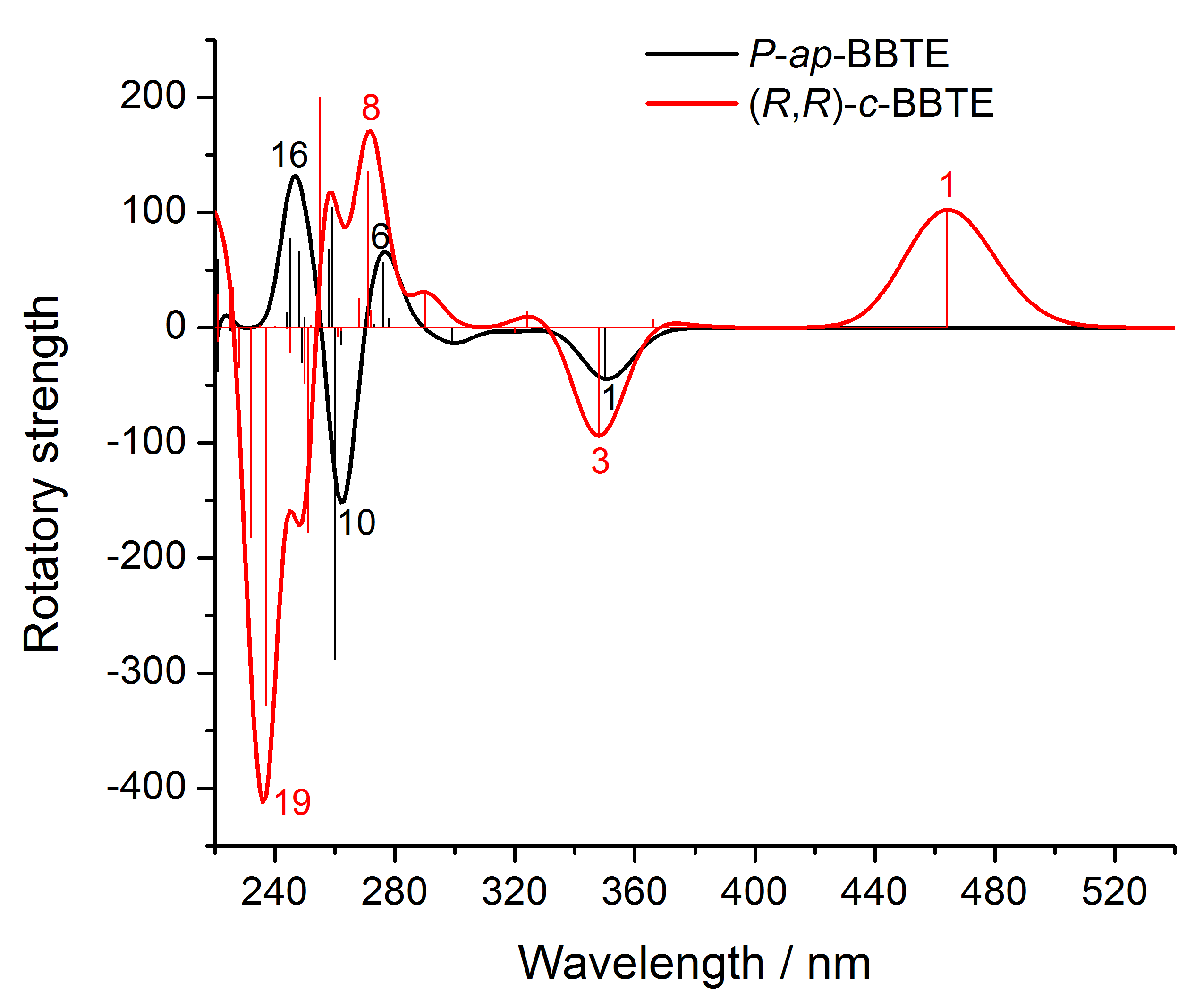


**Figure S6.** Computed (left) absorption spectra and (right) CD spectra of (*R,R*)-*c*-**BBTE** and *P*-*ap*-**BBTE**, at the CAM-B3LYP/6-311+G(d,p)/PCM level of theory.

**Table S1. Computed excitation energies, oscillator strengths and molecular orbital compositions of important low-lying excited singlet states, at the CAM-B3LYP/6-311+G(d,p)/PCM level of theory.**

| Compound | State | *λ*_abs_ | *f* | MO composition*^a^* |
| --- | --- | --- | --- | --- |
| *P*-*ap*-**BBTE** | S_1_ | 3.54 eV, 350 nm | 0.048 | H-0 → L+0 (93%) |
|  | S_3_ | 4.13 eV, 299 nm | 0.047 | H-0 → L+1 (63%)  H-4 → L+1 (22%) |
|  | S_4_ | 4.32 eV, 287 nm | 0.230 | H-4 → L+0 (72%)  H-2 → L+0 (15%) |
|  | S_6_ | 4.49 eV, 276 nm | 0.226 | H-1 → L+1 (74%)  H-5 → L+1 (16%) |
|  | S_9_ | 4.72 eV, 262 nm | 0.473 | H-5 → L+1 (64%)  H-1 → L+1 (17%) |
|  | S_10_ | 4.76 eV, 260 nm | 0.211 | H-0 → L+2 (35%)  H-5 → L+0 (18%)  H-1 → L+3 (14%) |
|  | S_16_ | 5.04 eV, 245 nm | 0.173 | H-0 → L+3 (24%)  H-3 → L+2 (16%)  H-3 → L+1 (15%) |
| (*R*,*R*)-*c*-**BBTE** | S_1_ | 2.67 eV, 464 nm | 0.180 | H-0 → L+0 (87%) |
|  | S_2_ | 3.38 eV, 366 nm | 0.242 | H-1 → L+0 (67%)  H-0 → L+2 (18%) |
|  | S_3_ | 3.56 eV, 348 nm | 0.172 | H-0 → L+1 (77%) |
|  | S_4_ | 3.82 eV, 324 nm | 0.154 | H-0 → L+2 (65%)  H-1 → L+0 (13%) |
|  | S_5_ | 3.86 eV, 320 nm | 0.125 | H-2 → L+0 (67%)  H-2 → L+1 (11%) |
|  | S_8_ | 4.56 eV, 271 nm | 0.255 | H-4 → L+0 (69%) |
|  | S_19_ | 5.22 eV, 237 nm | 0.117 | H-4 → L+1 (42%)  H-2 → L+1 (36%) |

Note: Molecular orbitals located at the benzobis(thiadiazole) bridge are marked in red.


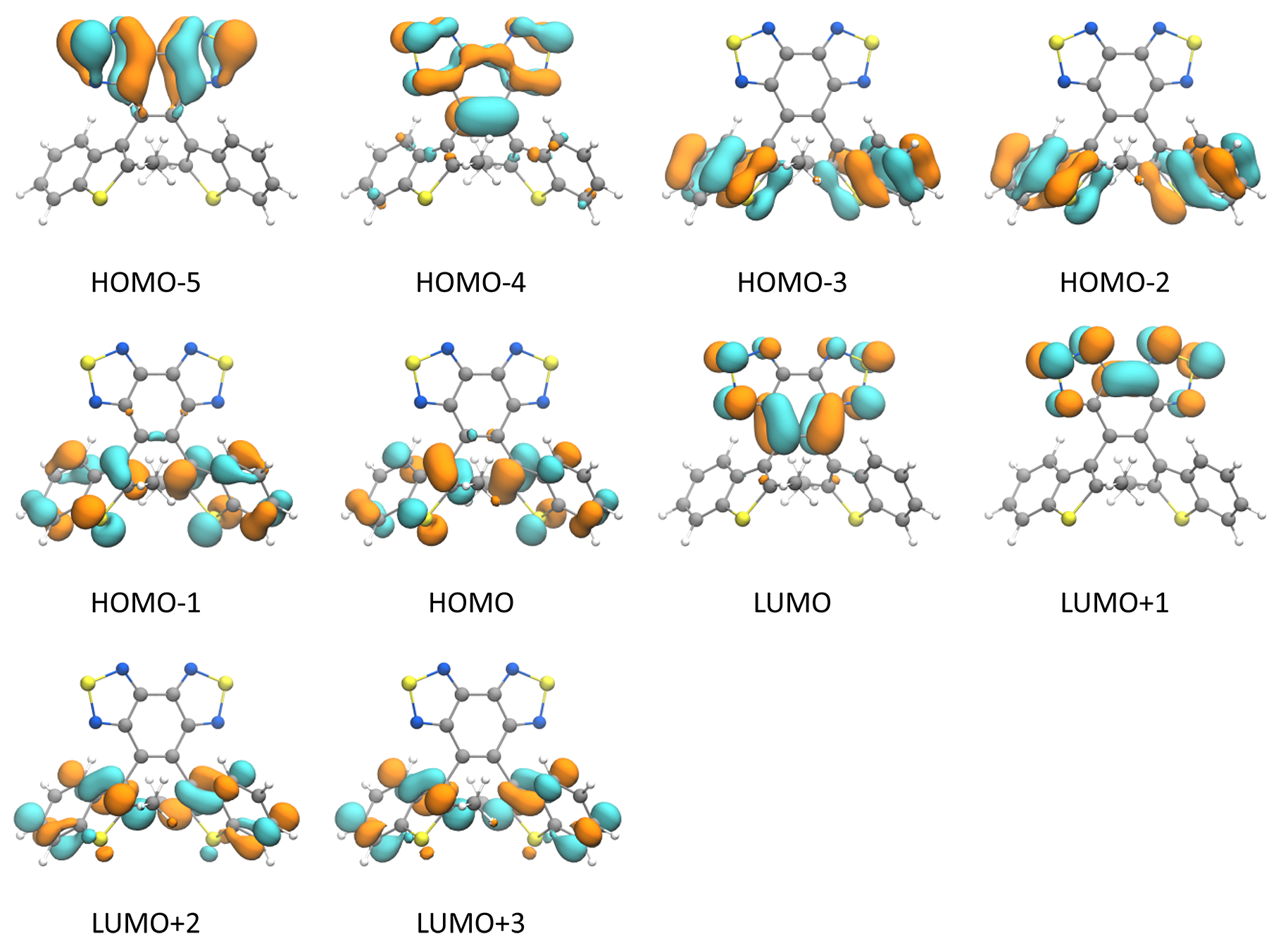


**Figure S7.** Frontier molecular orbitals of *P*-*ap*-**BBTE**.


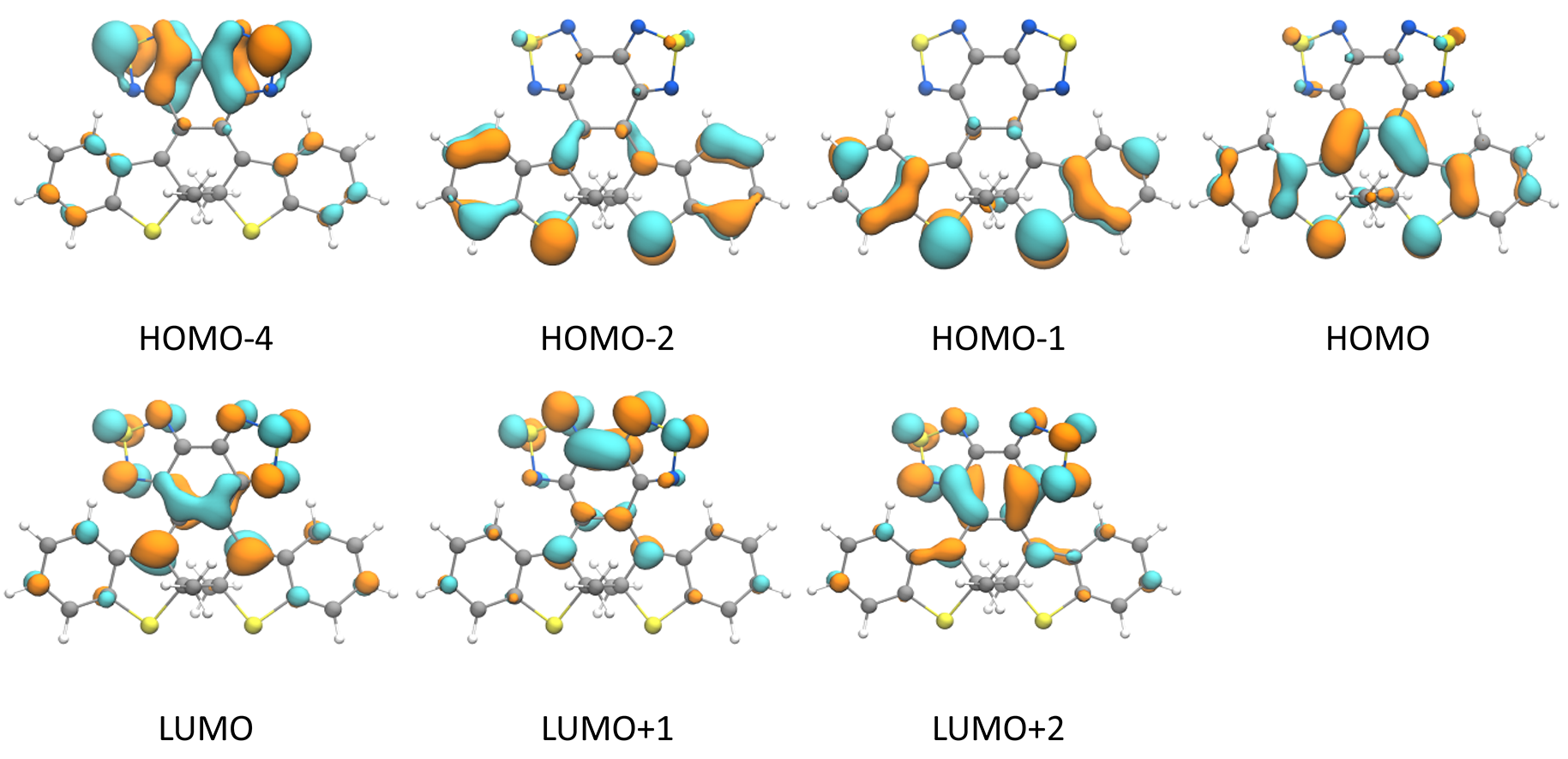


**Figure S8.** Frontier molecular orbitals of (*R*,*R*)-*c*-**BBTE**.

# 5. Crystal data

**Table S2. Crystal data of *P*-*ap*- and *M*-*ap***-BBTE

| Compounds | *M*-*ap*-**BBTE** | *P*-*ap*-**BBTE** |
| --- | --- | --- |
| CCDC Number | 1016417 | 1016418 |
| Empirical formula | C_24_H_14_N_4_S_4_ | C_24_H_14_N_4_S_4_ |
| Formula weight | 486.63 | 486.63 |
| Temperature / K | 140(2) | 293(2) |
| Crystal system | monoclinic | monoclinic |
| Space group | C2 | C2 |
| a / Å | 20.889(2) | 20.857(4) |
| b / Å | 6.8331(8) | 6.8324(13) |
| c / Å | 15.2581(17) | 15.250(4) |
| α / deg | 90 | 90 |
| β / deg | 98.869(2) | 98.853(2) |
| γ / deg | 90 | 90 |
| Volume / Å^3^ | 2151.9(4) | 2147.3(8) |
| Z | 4 | 4 |
| ρ_calc_ / mg/mm^3^ | 1.502 | 1.505 |
| F (000) | 1000.0 | 1000.0 |
| Crystal size / mm^3^ | 0.2 × 0.15 × 0.08 | 0.25 × 0.12 × 0.02 |
| 2Θ range for data collection / deg | 2.702 - 61.096 | 2.702 - 61.2 |
| Index ranges | -29 ≤ h ≤ 28,  -7 ≤ k ≤ 9,  -21 ≤ l ≤ 21 | -29 ≤ h ≤ 26,  -9 ≤ k ≤ 9,  -20 ≤ l ≤ 21 |
| Reflections collected | 11056 | 10967 |
| Independent reflections, R(int) | 5384, 0.0357 | 6516, 0.0379 |
| GOF on F^2^ | 1.008 | 1.016 |
| Final R indexes [I ≥ 2σ (I)] | R_1_ = 0.0373, wR_2_ = 0.0856 | R_1_ = 0.0479, wR_2_ = 0.1071 |
| Final R indexes [all data] | R_1_ = 0.0505, wR_2_ = 0.0994 | R_1_ = 0.0637, wR_2_ = 0.1228 |
| Largest diff. peak / hole / e Å^-3^ | 0.39 / -0.41 | 0.48 / -0.41 |
| Flack parameter | 0.03(5) | 0.00(6) |

**Table S3. Crystal data of (*S,S*)-*c*- and (*R,R*)-*c***-BBTE

| Compounds | (*S,S*)-*c*-**BBTE** | (*R,R*)-*c*-**BBTE** |
| --- | --- | --- |
| CCDC Number | 1016420 | 1016419 |
| Empirical formula | C_24_H_14_N_4_S_4_ | C_24_H_14_N_4_S_4_ |
| Formula weight | 486.63 | 486.63 |
| Temperature / K | 140(2) | 140(2) |
| Crystal system | orthorhombic | orthorhombic |
| Space group | P2_1_2_1_2_1_ | P2_1_2_1_2_1_ |
| a / Å | 9.1365(7) | 9.1216(9) |
| b / Å | 13.9691(11) | 13.9119(13) |
| c / Å | 16.1282(13) | 16.1175(15) |
| α / deg | 90 | 90 |
| β / deg | 90 | 90 |
| γ / deg | 90 | 90 |
| Volume / Å^3^ | 2058.4(3) | 2045.3(3) |
| Z | 4 | 4 |
| ρ_calc_ / mg/mm^3^ | 1.570 | 1.580 |
| F (000) | 1000.0 | 1000.0 |
| Crystal size / mm^3^ | 0.4 × 0.32 × 0.28 | 0.35 × 0.29 × 0.2 |
| 2Θ range for data collection / deg | 3.858 - 61.248 | 3.868 - 61.232 |
| Index ranges | -13 ≤ h ≤ 13,  -16 ≤ k ≤ 19,  -23 ≤ l ≤ 21 | -12 ≤ h ≤ 13,  -19 ≤ k ≤ 19,  -19 ≤ l ≤ 23 |
| Reflections collected | 20949 | 20837 |
| Independent reflections, R(int) | 6302, 0.0417 | 6268, 0.0388 |
| GOF on F^2^ | 0.992 | 1.033 |
| Final R indexes [I ≥ 2σ (I)] | R_1_ = 0.0333, wR_2_ = 0.0776 | R_1_ = 0.0354, wR_2_ = 0.0884 |
| Final R indexes [all data] | R_1_ = 0.0387, wR_2_ = 0.0802 | R_1_ = 0.0394, wR_2_ = 0.0907 |
| Largest diff. peak / hole / e Å^-3^ | 0.26 / -0.35 | 0.38 / -0.46 |
| Flack parameter | 0.01(3) | -0.02(2) |


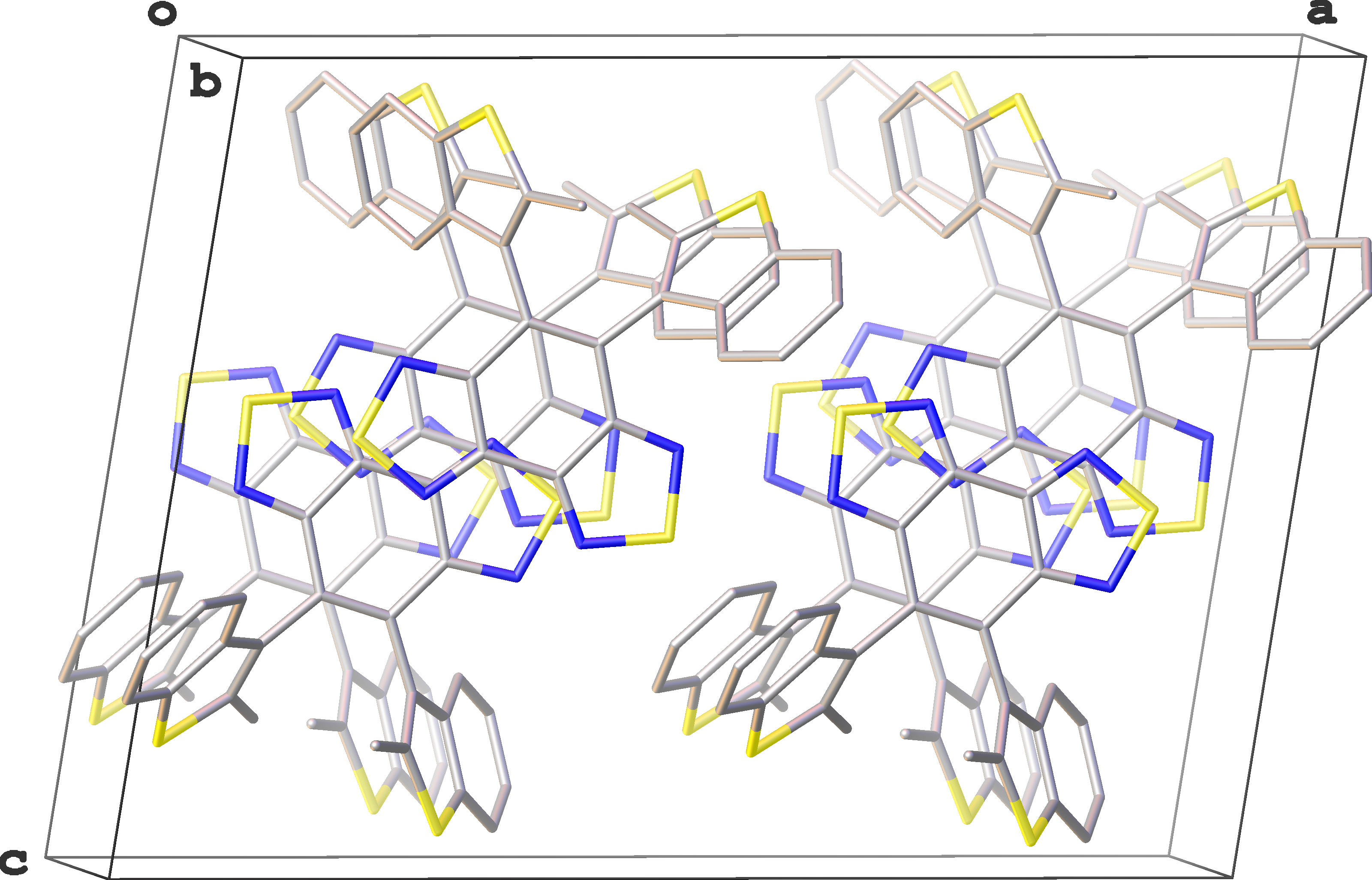


**Figure S9**. Packing diagram of *M*-*ap*-**BBTE** (S: yellow; N: blue; C: gray). Atoms with lighter colours indicate their relatively back location


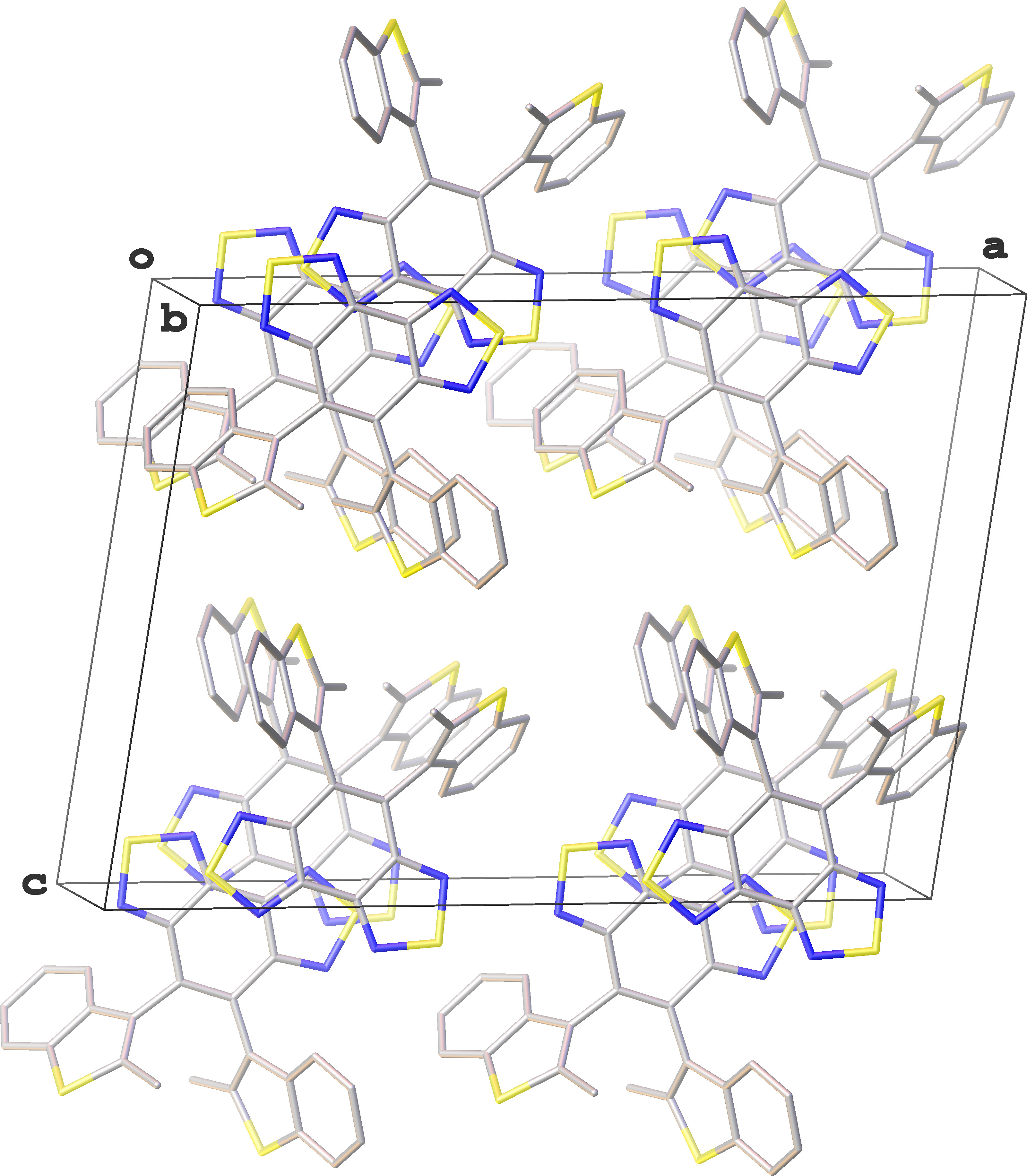


**Figure S10**. Packing diagram of *P*-*ap*-**BBTE** (S: yellow; N: blue; C: gray). Atoms with lighter colours indicate their relatively back location

**
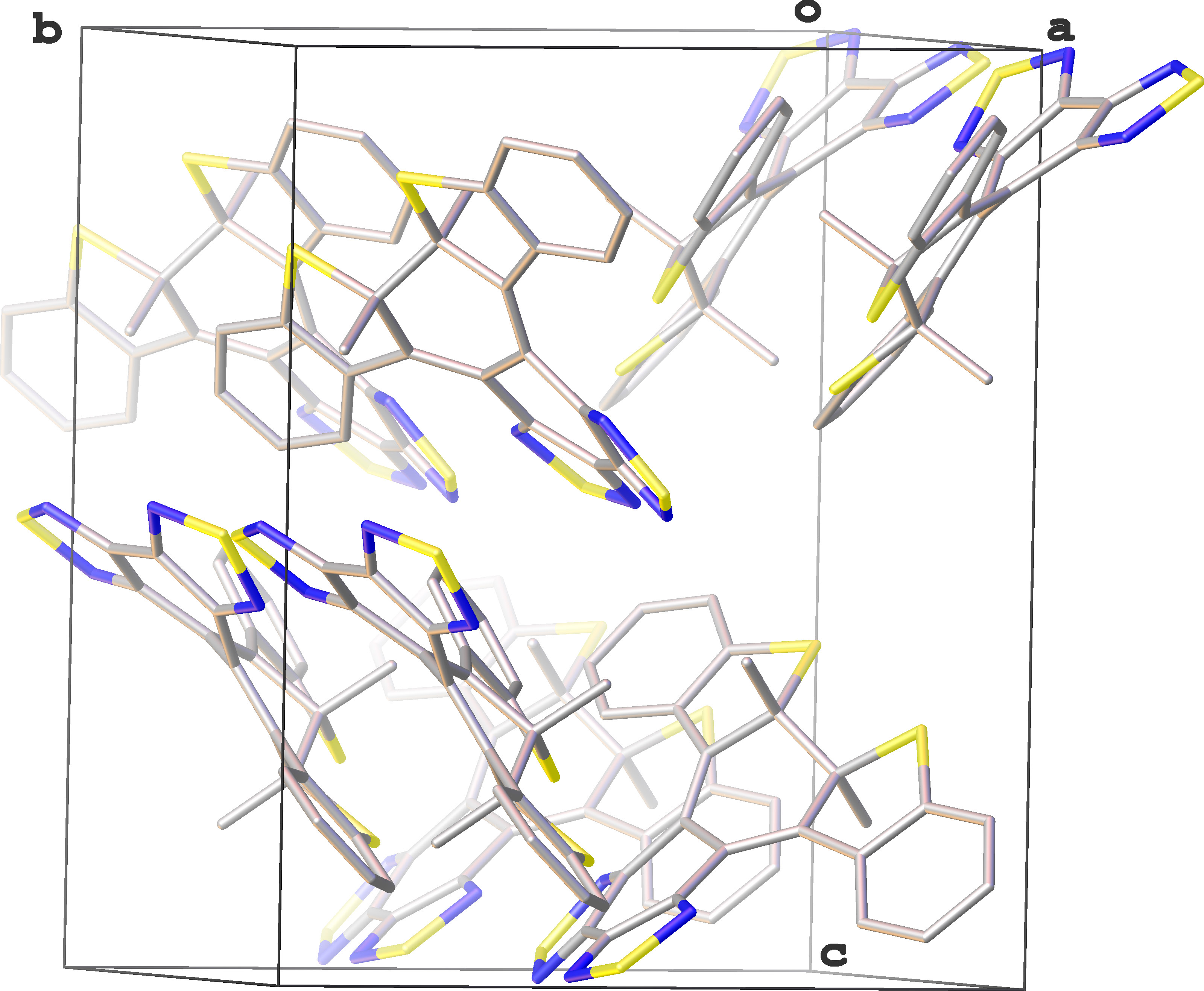
**

**Figure S11**. Packing diagram of *(S,S)-c-***BBTE** (S: yellow; N: blue; C: gray). Atoms with lighter colours indicate their relatively back location


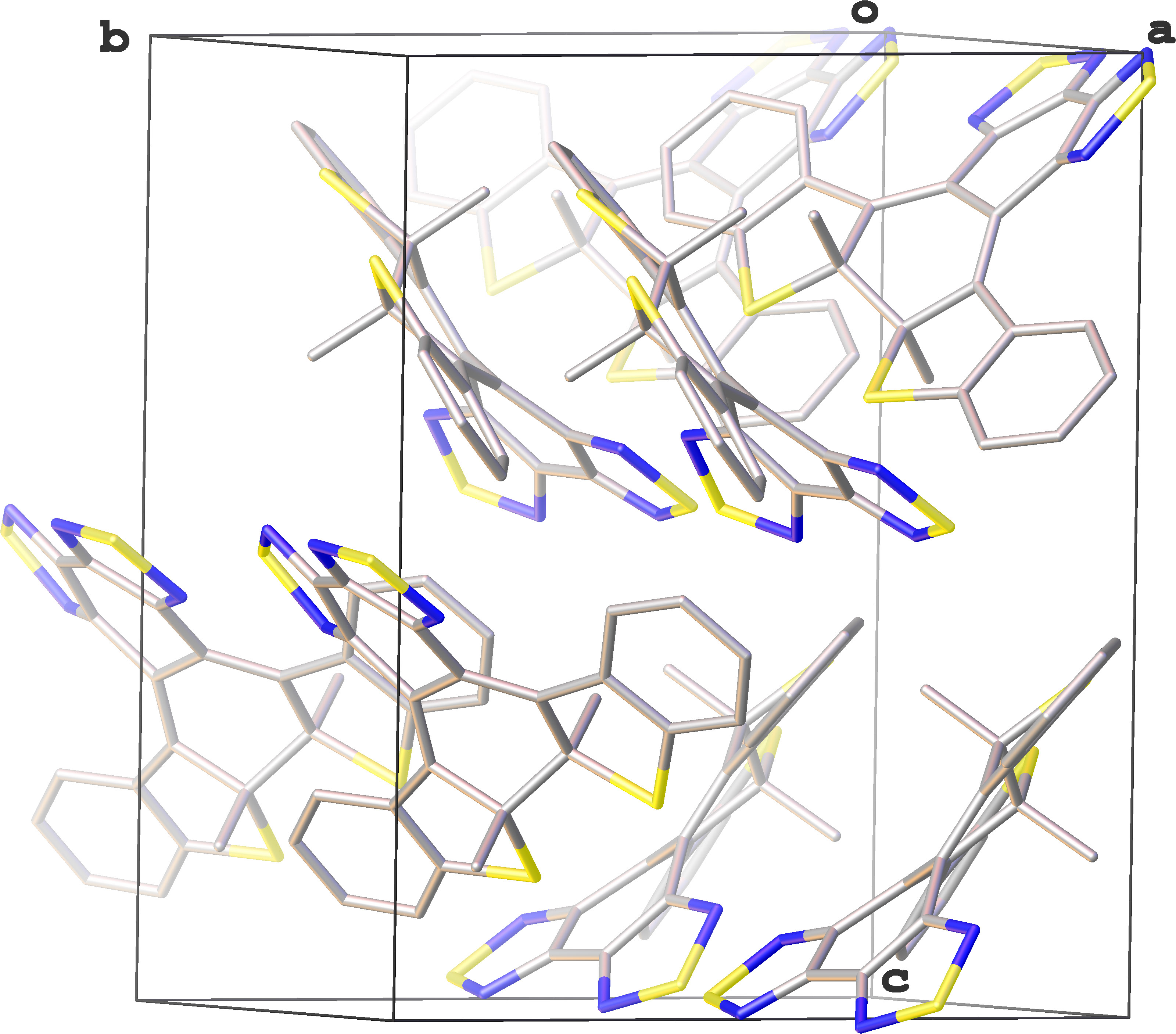


**Figure S12**. Packing diagram of *(R,R)-c-***BBTE** (S: yellow; N: blue; C: gray). Atoms with lighter colours indicate their relatively back location

# 6. Characterization


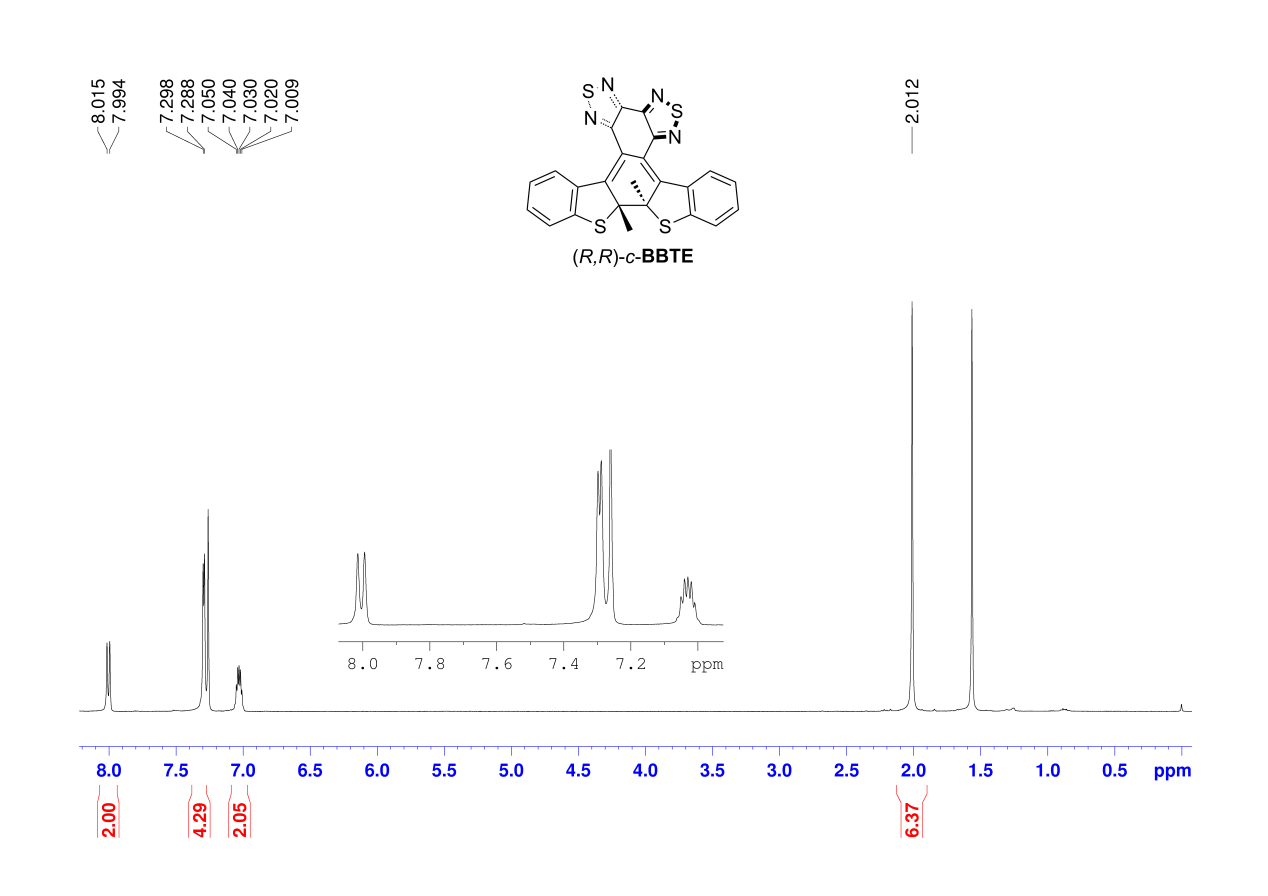


**Figure S13**. ^1^H NMR spectrum of (*R,R*)-*c*-**BBTE** in CDCl_3_.


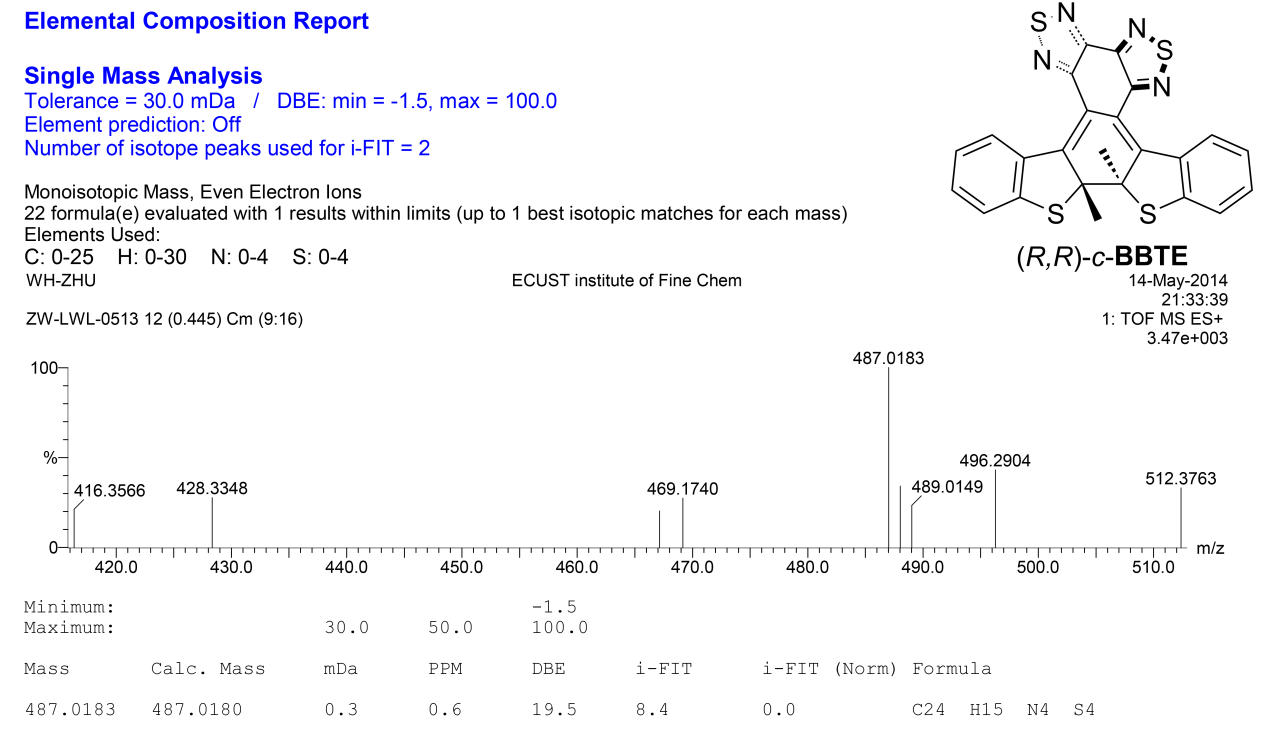


**Figure S14**. HRMS spectrum of (*R,R*)-*c*-**BBTE**.


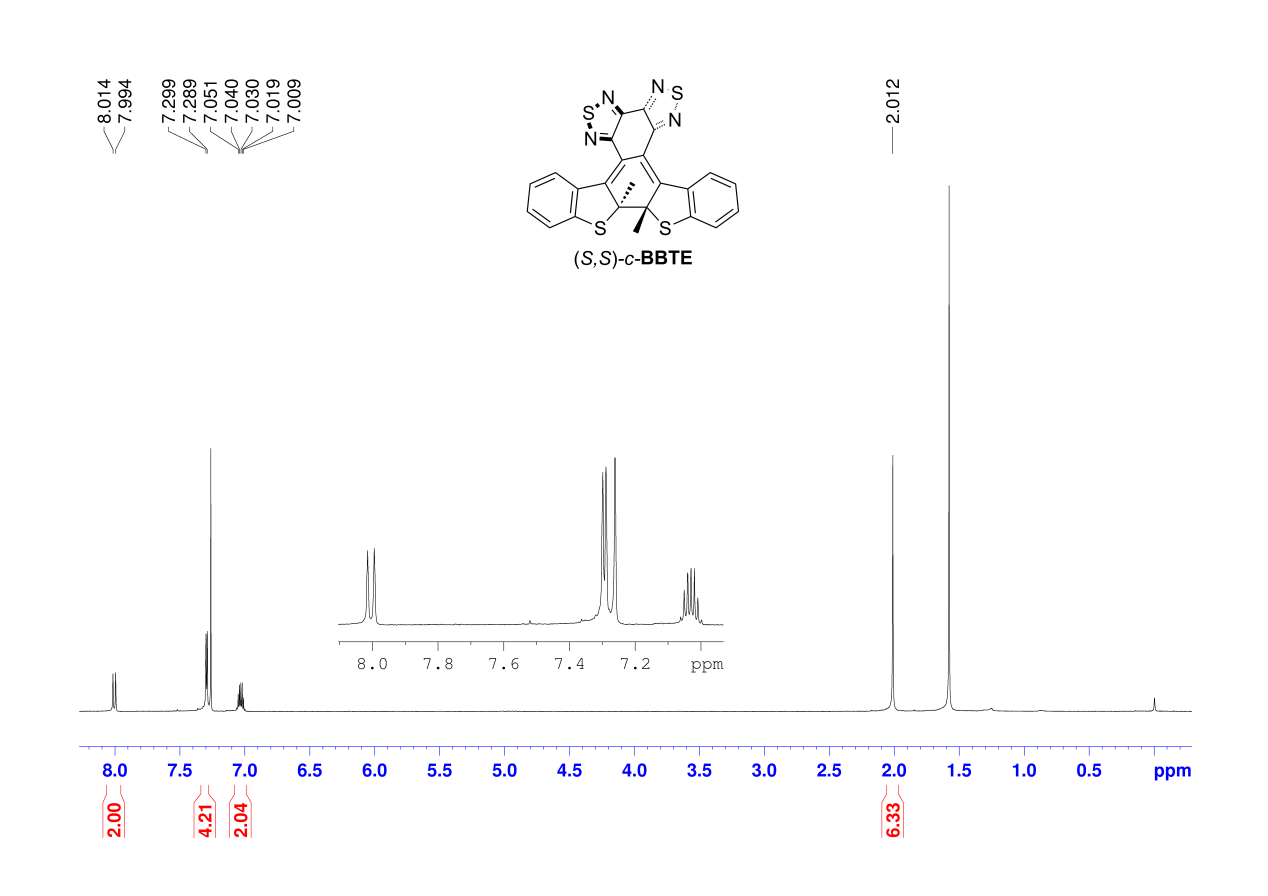
**Figure S15**. ^1^H NMR spectrum of (*S,S*)-*c*-**BBTE** in CDCl_3_.


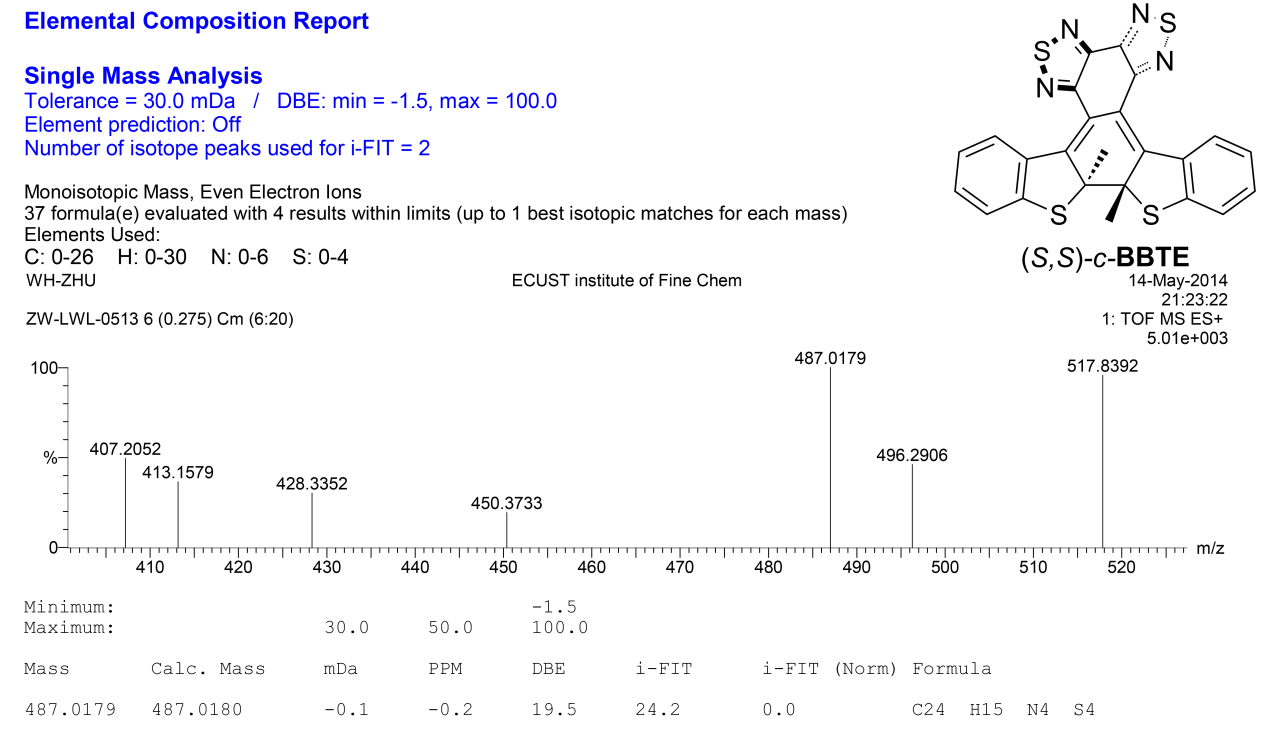


**Figure S16**. HRMS spectrum of (*S,S*)-*c*-**BBTE**.


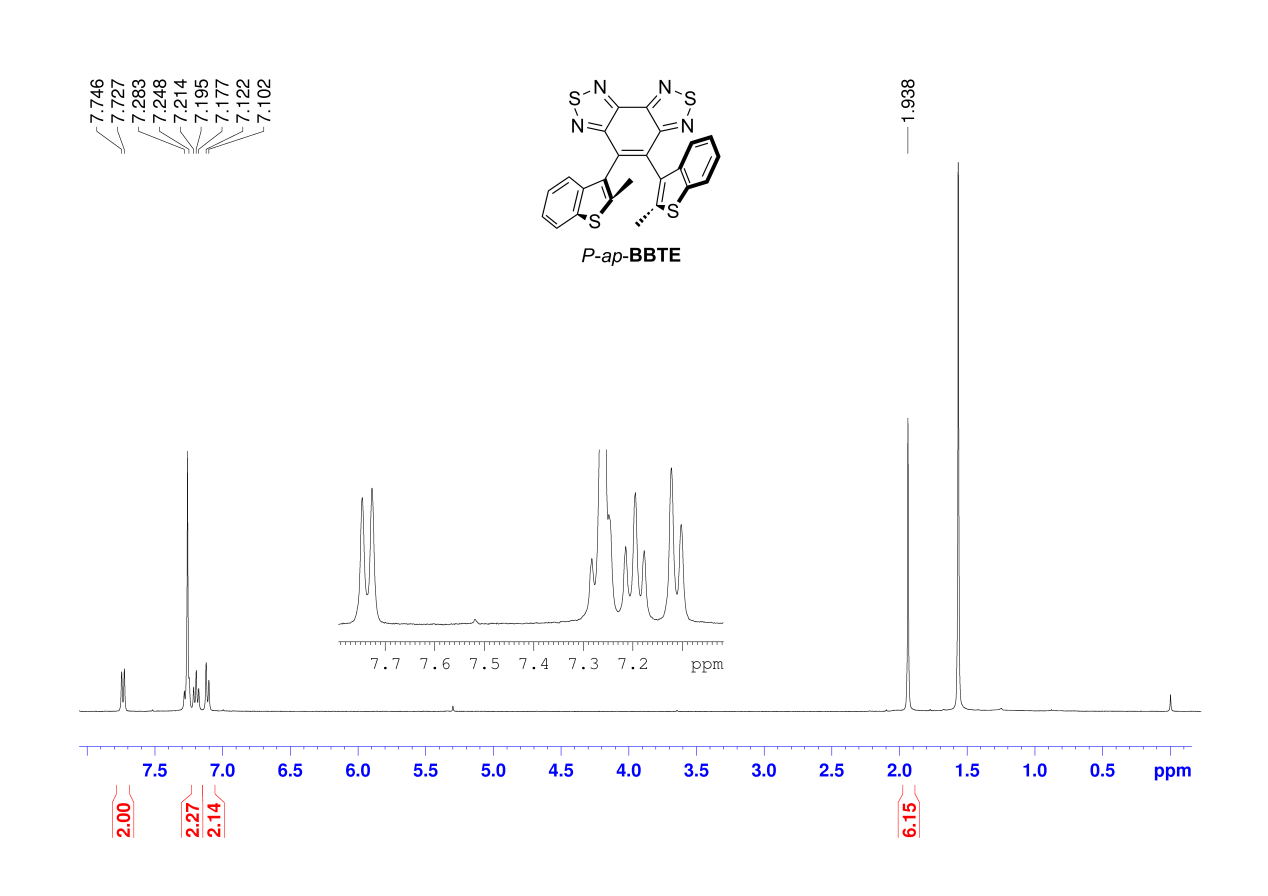


**Figure S17**. ^1^H NMR spectrum of *P*-*ap*-**BBTE** in CDCl_3_.


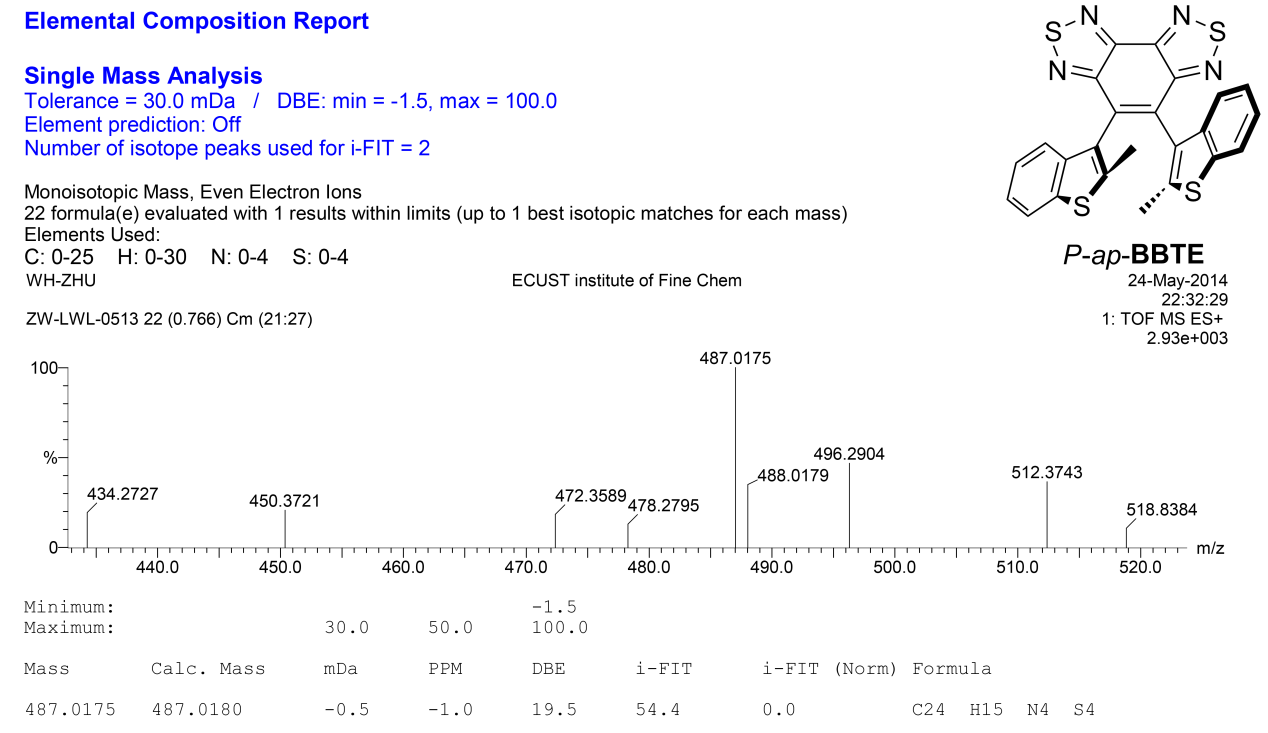


**Figure S18**. HRMS spectrum of *P*-*ap*-**BBTE**.


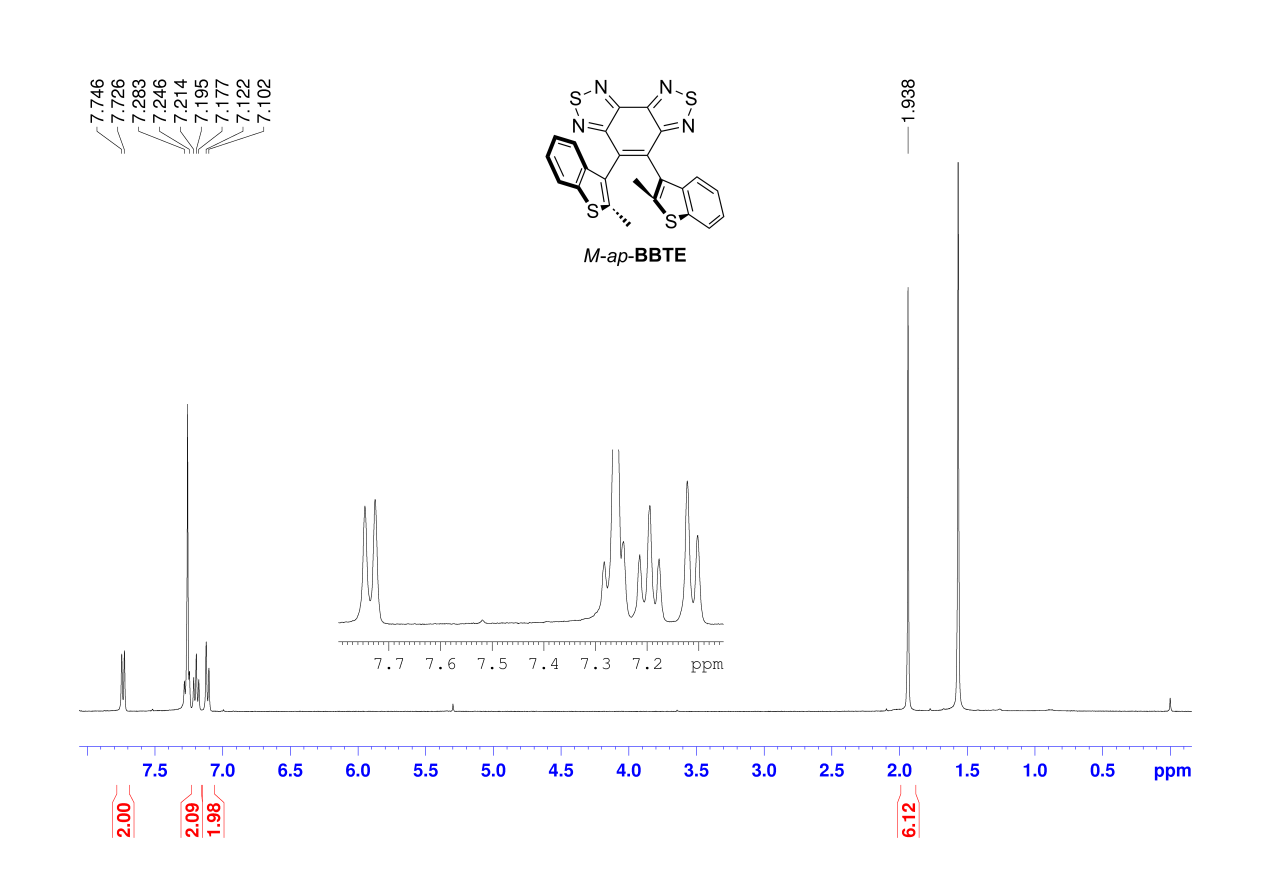


**Figure S19*.*** ^1^H NMR spectrum of *M*-*ap*-**BBTE** in CDCl_3_.


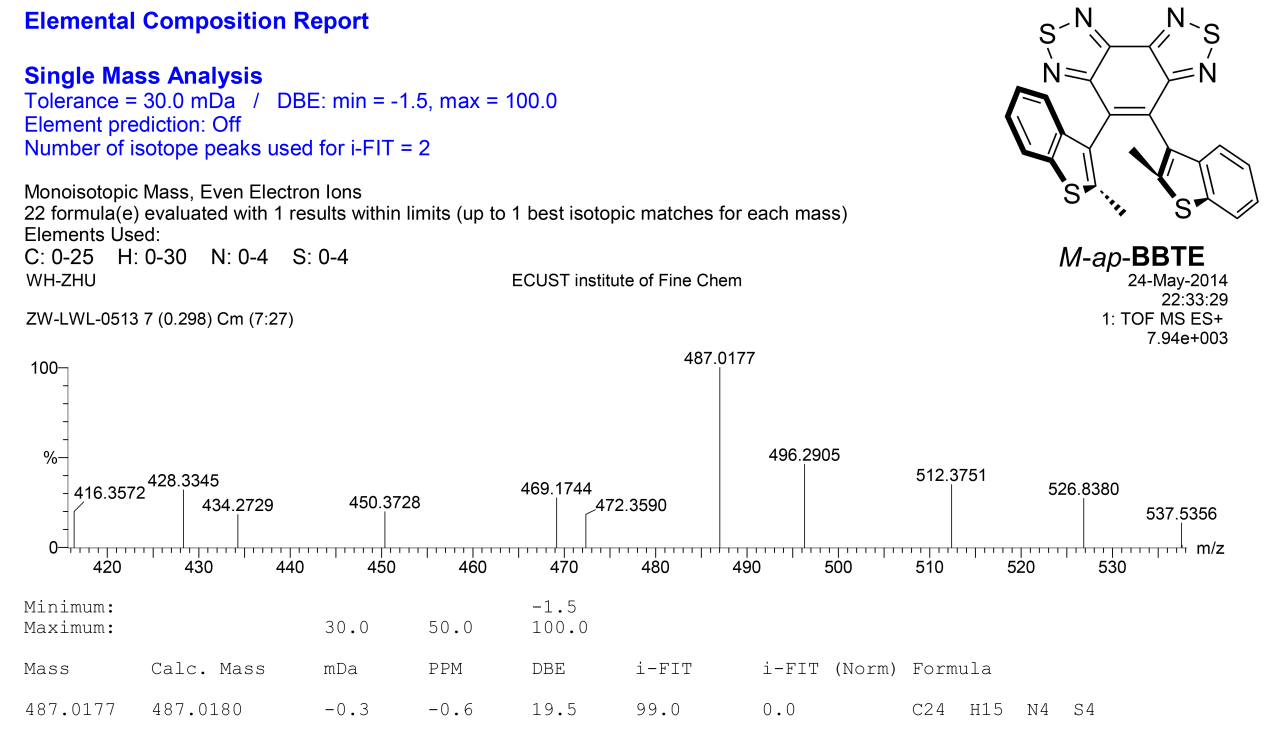


**Figure S20*.*** HRMS spectrum of *M*-*ap*-**BBTE**.
